# Formation of Strong Boron Lewis Acid Sites on Silica

**DOI:** 10.1021/acs.inorgchem.3c04121

**Published:** 2024-03-07

**Authors:** Kavyasripriya K. Samudrala, Manjur O. Akram, Jason L. Dutton, Caleb D. Martin, Matthew P. Conley

**Affiliations:** † Department of Chemistry, University of California, Riverside, California 92521, United States; ‡ Department of Chemistry and Biochemistry, Baylor University, Waco, Texas 76798, United States; § Department of Biochemistry and Chemistry, La Trobe Institute for Molecular Science, La Trobe University, Melbourne, Victoria 3086, Australia

## Abstract

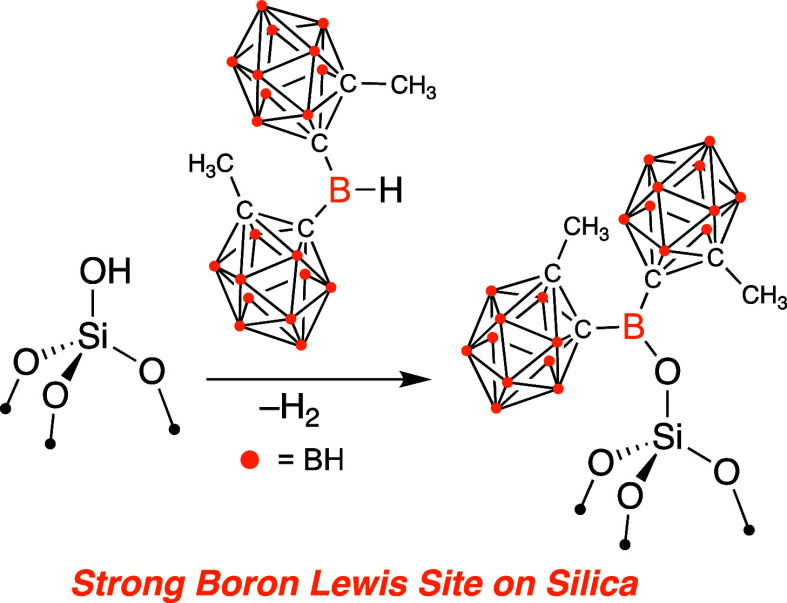

Bis(1-methyl-*ortho*-carboranyl)borane (HB^Me^*o*Cb_2_) is a very strong Lewis acid that reacts with the isolated silanols present on silica partially dehydroxylated at 700 °C (SiO_2-700_) to form the well-defined Lewis site ^Me^*o*Cb_2_B(OSi≡) (**1**) and H_2_. ^11^B{^1^H} magic-angle spinning (MAS) nuclear magnetic resonance (NMR) data of **1** are consistent with that of a three-coordinate boron site. Contacting **1** with O=PEt_3_ (triethylphosphine oxide TEPO) and measuring ^31^P{^1^H} MAS NMR spectra show that **1** preserves the strong Lewis acidity of HB^Me^*o*Cb_2_. Hydride ion affinity and fluoride ion affinity calculations using small molecules analogs of **1** also support the strong Lewis acidity of the boron sites in this material. Reactions of **1** with Cp_2_Hf(^13^CH_3_)_2_ show that the Lewis sites are capable of abstracting methide groups from Hf to form [Cp_2_Hf–^13^CH_3_][H_3_^13^C–B(^Me^*o*Cb_2_)OSi≡], but with a low overall efficiency.

## Abstract 2

Bis(1-methyl-*ortho*-carboranyl)borane (HB^Me^*o*Cb_2_) is a very strong Lewis acid that reacts with the isolated silanols present on silica partially dehydroxylated at 700 °C (SiO_2-700_) to form the well-defined Lewis site ^Me^*o*Cb_2_B(OSi≡) (**1**) and H_2_.

## Introduction

The interface of materials science and organometallic chemistry is a rich landscape for the development and application of well-defined heterogeneous catalysts for a variety of chemical transformations.^1^ This field depends on the discrete understanding of surface sites present on a material, typically a high surface area oxide, and how those sites react with an organometallic. Nearly all oxides are terminated with –OH sites that react with organometallics through protonolysis reactions, shown in eq 1 between a generic organometallic and surface hydroxyl to generate either L_n_M–O_X_ or L_n_M···O_X_ ion-pairs (O_X_ = surface oxygen). The type of surface site formed in this reaction usually depends on the acidity of the surface hydroxyl,^2^ which is dependent on the type of oxide used and is often encountered when using less common oxide supports.^3^
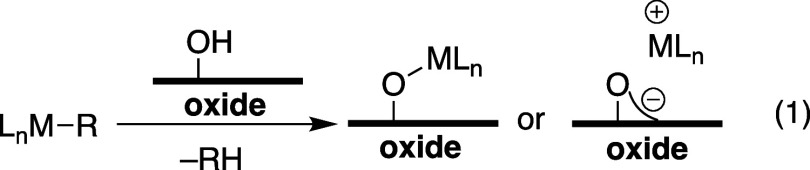
1

**Figure 1:**
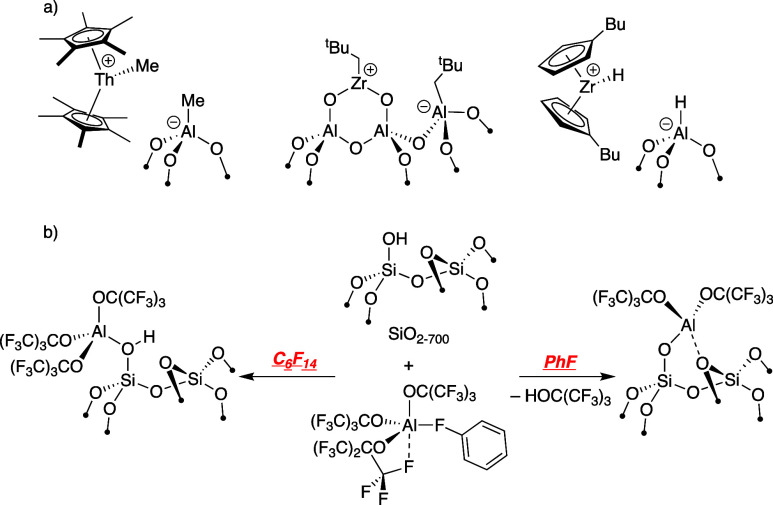
Representative examples of ion pairs formed on Al_2_O_3_.

Alumina (Al_2_O_3_) is a classic example, where the oversimplification shown in eq 1 breaks down. Typical γ-Al_2_O_3_ materials are also terminated with −OH groups, but a small quantity of strong Lewis sites persists on these materials.^4^ The Lewis sites play an important role in the formation of catalytically active sites on alumina, Figure 1a. For example, Al_2_O_3_ dehydrated at 1000 °C reacts with Cp*_2_Th(CH_3_)_2_ (Cp* = pentamethylcyclopentadienyl) to generate [Cp*_2_Th–CH_3_][H_3_C–AlO_X_] formed by methide abstraction by Lewis acidic Al-sites.^5^ Lower dehydroxylation temperatures also preserve this type of reactivity, exemplified by the reaction of Al_2_O_3_ partially dehydroxylated at 500 °C with Zr(CH_2_^*t*^Bu)_4_ to form [Zr(CH_2_^*t*^Bu)(O_X_)_2_][^*t*^BuH_2_C–AlO_X_].^6^ Both results are related to the reactivity of common olefin polymerization compositions containing metallocenes, AlR_3_, and Al_2_O_3_ that form [Cp^b^_2_Zr–H][H–AlO_X_] ion-pairs (Cp^b^ = 1-butylcyclopentadienyl).^7^

Each of the examples in Figure 1a rely on the exceedingly low surface coverage of the Lewis site present on dehydrated aluminas.^4b^ Generating strong Lewis sites on oxides using the reaction shown in eq 1 with alkylaluminum^8,9^ or alkylgallium^10^ tends to form mixtures of surface species. Al(OC(CF_3_)_3_)(PhF),^11^ a very strong Lewis acid, shows solvent-dependent reactivity with silica partially dehydroxylated at 700 °C (SiO_2-700_) shown in Figure 1b. In perfluorohexane, Al(OC(CF_3_)_3_)(PhF) reacts with the isolated silanols present on SiO_2-700_ to form Brønsted acidic bridging silanols that behave as weakly coordination anions when deprotonated.^12^ In fluorobenzene, Al(OC(CF_3_)_3_)(PhF) reacts with SiO_2-700_ in the more traditional manner shown in eq 1.^13^ These aluminum sites participate in similar alkyl abstraction reactions as those shown in Figure 1.^13,14^

Access to oxides containing boron Lewis sites continues to be challenging. Common boric acid impregnation methods followed by heat treatment tend to form networks containing mixtures of 3- or 4-coordinate boron from solid-state NMR studies.^15,16^ Though lacking strong Lewis acidity, many of these materials show interesting reactivity in oxidative dehydrogenation reactions.^17^ BCl_3_ or BF_3_ also react with silica and probably form poorly defined Lewis sites.^18^

**Figure 2:**
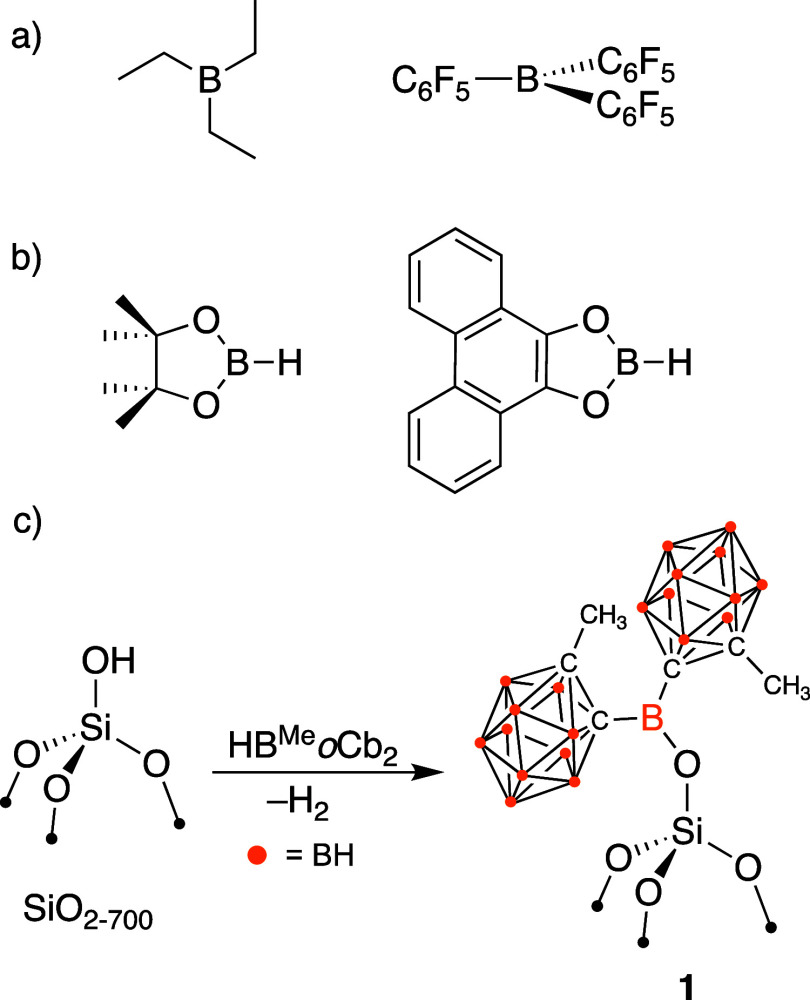
BR_3_ (a) or HBR_2_ (b) reagents used in silica functionalization reactions. The reaction of bis(1-methyl-*ortho*-carboranyl)borane (HB^Me^*o*Cb_2_) with SiO_2-700_ is described in this study (c).

Reactions of trialkylboranes to generate Lewis sites are limited to the examples shown in Figure 2a. BEt_3_ reacts with partially dehydroxylated silicas and is claimed to form Et_2_B–OSi≡ from Fourier transform infrared (FTIR) studies.^19^ B(C_6_F_5_)_3_, a common strong Lewis acid,^20^ forms adducts with SiO_2-700_ that can be trapped in the presence of *N*,*N*-dimethylaniline to form supported anilinium sites,^21,22^ but direct protonation by surface silanols to form the Lewis acidic (C_6_F_5_)_2_B–OSi≡ and C_6_F_5_H was not observed. Silica dehydrated at 500 °C (SiO_2-500_) results in the formation of pairs of Lewis sites that involves the adsorbed water on SiO_2-500_ and forms pairs of (C_6_F_5_)_2_B–OSi≡.^23^ However, these Lewis sites are not sufficiently acidic to abstract a methide from Cp_2_Zr(CH_3_)_2_. Related species were studied in solution using isolable silsesquioxanes as models for silica surfaces.^24^

HBR_2_ tends to be more reactive toward silica.^25,26^ Pinacolborane reacts with silica to form well-defined pinacolborate species that were studied in detail by solid-state nuclear magnetic resonance (NMR) spectroscopy.^27^ Phenantro[9,10-d][1,3,2]dioxaborole reacts with SiO_2-700_ either by elimination of H_2_ or by protonolysis of a B–O group to form well-defined surface borates.^28^ Lewis acidity was not studied in these examples, but B(OR)_3_ species, even when containing perfluorinated alkoxy groups, are mild Lewis acids.^29^

This paper describes the reaction of bis(1-methyl-*ortho*-carboranyl)borane (HB^Me^*o*Cb_2_)^30^ with SiO_2-700_, Figure 2c. HB^Me^*o*Cb_2_ is a Lewis superacid, defined as Lewis acids having higher fluoride ion affinity (FIA) than that of SbF_5_.^29,31^ The *ortho*-carboranyl functionalities attached to the central boron act as strong electron-withdrawing groups and provide a more congested steric environment around the Lewis acidic boron compared to that of −C_6_F_5_.^32^ In addition, the Lewis acidic p-orbital on boron is highly localized on the central boron atom,^33^ which is in contrast to the more delocalized lowest unoccupied molecular orbital (LUMO) in B(C_6_F_5_)_3_. The data described below show that the boron sites formed in reactions with silica that generate **1** are very Lewis acidic. As an entry point to this study, we also describe the reactions that generate HO–B^Me^*o*Cb_2_ (**2**) and Me_3_Si–O–B^Me^*o*Cb_2_ (**3**), both of which serve as rough molecular analogs of **1**.

## Experimental Section

### General Considerations

All manipulations were performed under an inert atmosphere of dinitrogen or argon using standard Schlenk or glovebox techniques. Benzene-*d*_6_ was purchased from Cambridge Isotope Laboratories, dried over sodium/benzophenone, degassed by consecutive freeze–pump–thaw cycles, distilled under a vacuum, and stored in an inert atmosphere glovebox. FTIR spectra were recorded in transmission mode as pressed pellets using a Bruker Alpha IR spectrometer in an argon-filled glovebox. HB^Me^*o*Cb_2_ and BrB^Me^*o*Cb_2_ were prepared according to literature procedures.^30^ Multinuclear NMR spectra (^1^H, ^13^C{^1^H}, and ^11^B{^1^H}) were recorded on a Bruker AVANCE III HD 400 or 600 MHz instrument. All solid-state NMR samples were packed in 4 mm zirconia rotors and sealed with a Kel-F cap under an argon or dinitrogen atmosphere in a glovebox. Solid-state NMR spectra were recorded under magic-angle spinning (MAS) or under static conditions at 14.1 T using Bruker NEO600 spectrometer. All solid-state NMR processing used Bruker Topspin. Single-crystal X-ray diffraction data were collected on a Bruker Apex III-CCD detector using Mo–Kα radiation (λ = 0.71073 Å). Crystals were selected under paratone oil, mounted on MiTeGen micromounts, and immediately placed in a cold stream of N_2_. Structures were solved and refined using SHELXTL, and figures were produced using OLEX2.

#### Synthesis of **1**

SiO_2-700_ (0.5 g, 0.13 mmol OH) and HB^Me^*o*Cb_2_ (0.040 g, 0.13 mmol, 1.0 mol equiv) were transferred to one arm of a double-Schlenk flask inside an argon-filled glovebox. The flask was removed from the glovebox, connected to a high vacuum line, and evacuated for 5 min. Benzene (∼6 mL) was condensed onto the solids at 77 K. The mixture warmed to room temperature and stirred for 30 min. During this time, the mixture was stirred gently to promote mixing and prevent the compacted silica from breaking into smaller fragments. After this time, the clear colorless solution was filtered away from **1** to the other side of the double Schlenk. The arm of the double Schlenk containing **1** was cooled to 0 °C, causing the benzene on the other side of the flask to condense onto the solid. The benzene was warmed to 25 °C, stirred for 5 min, and filtered back to the other side of the double Schlenk. This procedure was repeated two more times to wash any residual HB^Me^*o*Cb_2_ away from **1**. The volatiles from the reaction mixture were analyzed by gas chromatography–thermal conductivity detector (GC–TCD) (Figure S1 in the Supporting Information) resulting in 0.23 mmol of H_2_/g of SiO_2_. **1** was dried under vacuum for 40 min. The white H-BSO solid was stored in an Ar glovebox freezer at −20 °C. Inductively coupled plasma–optical emission spectrometry (ICP–OES) of **1** after digestion in 5% nitric acid solution gives 4.77 mmol_B_ g^–1^. Cross-polarization MAS carbon NMR (^13^C{^1^H} CPMAS NMR) data: 25 (≡Si–O–B(**Me***o*Cb)_2_), 71, and 78 ppm (≡Si–O–B(Me*o***Cb**)_2_). ^11^B{^1^H} MAS NMR data: 33, 2, −6, −8, and −11 ppm, respectively.

#### Synthesis of 1*TEPO

An essentially identical procedure for **1** was used to generate 1*TEPO. **1** (0.2 g, 0.046 mmol Lewis acidic B), triethylphosphine oxide (TEPO) (0.005 g, 0.9 equiv, 0.041 mmol), and pentane (∼5 mL) were used in this procedure. 1*TEPO was collected as a white solid and was stored in an Ar glovebox freezer at −20 °C. ^31^P{^1^H} MAS NMR data: 78 ppm (1*TEPO and 54 ppm (physisorbed TEPO). ^11^B{^1^H} MAS NMR data: 2, −1.5 ((≡Si–O–**B** (MeoCb)_2_(TEPO)), −6, −8, and −11 ppm. This spectrum contains a minor signal at 33 ppm from unreacted **1**.

#### Synthesis of 1*Hf

An essentially identical procedure to that for **1** was used to generate 1*Hf. **1** (0.100 g, 0.023 mmol Lewis acidic B), Cp_2_Hf(^13^CH_3_)_2_ (0.009 g, 1.1 equiv, 0.025 mmol), and pentane (∼5 mL) were used in this procedure. 1*Hf was collected as a white solid and was stored in an Ar glovebox freezer at −20 °C. The volatiles from the reaction mixture were analyzed by GC resulting in 0.07 mmol of CH_4_/g of SiO_2_. ICP–OES of 1 after digestion in 5% nitric acid solution gives 0.076 mmol_Hf_ g^–1^ and 5.03 mmol_B_ g^–1^. ^13^C{^1^H} CPMAS NMR data: 107.2 (Cp), 40.8 (Hf–^13^CH_3_ cation), 21.4 (Hf–^13^CH_3_ neutral), and 2.2 ppm (Si–^13^CH_3_). ^11^B{^1^H} MAS NMR data: 33 ppm (≡Si–O–**B** (Me*o*Cb)_2_), −1 ppm (≡Si–O–**B** (CH_3_)(Me*o*Cb)_2_), −6, −8, and −11 ppm.

#### Synthesis of HO–B^Me^oCb_2_ (**2**)

A solution of BrB^Me^*o*Cb_2_ (1.60 mmol, 650 mg) in chloroform (10 mL) was stirred in a vial, and neat Me_3_SiOH (1.60 mmol, 172.0 μL) was slowly added via micropipette at 23 °C. The reaction mixture was monitored by ^1^H and ^11^B NMR spectroscopy, and after 2 h, the reaction was complete. The volatiles were removed under reduced pressure to form a white solid, which was subsequently washed with pentane (2 × 2 mL). The white residue was dried under vacuum to give HOB^Me^*o*Cb_2_ as a white solid. Yield: 85%, 464 mg; ^1^H NMR (600 MHz, CDCl_3_): δ = 7.52 (s, 1H), 3.21–1.64 (m, 26H) ppm; ^13^C{^1^H} NMR (151 MHz, CDCl_3_): δ = 78.0, 26.0 ppm; ^11^B{^1^H} NMR (193 MHz, CDCl_3_): δ = 37.9 (s), 2.6 (s), −5.0 (s), −7.3 (s), −8.4 (s), −9.8 (s), −10.5 (s) ppm; HRMS(−ESI): calcd 361.3895 for C_6_H_26_B_21_O [M – H]^−^ found 361.3905.

#### Preparation of Me_3_SiOB^Me^oCb_2_ (**3**)

KH (0.150 mmol, 8.0 mg) was added in one portion to a solution of HOB^Me^*o*Cb_2_ (0.100 mmol, 34.2 mg) in Et_2_O (1 mL). The reaction mixture was then stirred at room temperature (23 °C) for 2 h. Excess solid KH was removed by filtration, and the solid was washed with Et_2_O (2 × 2 mL). The filtrate was reduced to ∼2 mL under vacuum, and trimethylsilyl trifluoromethanesulfonate (0.110 mmol, 20.0 μL) was added dropwise at room temperature (23 °C). The reaction was stirred at 23 °C for 30 min. After completion of the reaction, the volatiles were removed under vacuum, and the product was extracted using 1 mL cold toluene. The toluene was dried under vacuum to give the desired Me_3_Si–O–B^Me^*o*Cb_2_ as an unstable viscous liquid. Using this procedure, **3** was isolated in ∼95% purity. Yield: 29%, 12.0 mg; ^1^H NMR (600 MHz, CDCl_3_): δ = 7.37 (s, 1H), 2.75–1.87 (m, 26H), 0.46 (s, 9H) ppm; ^13^C{^1^H} NMR (151 MHz, CDCl_3_): δ = 77.6, 25.8, 2.3 ppm; ^11^B{^1^H} NMR (193 MHz, CDCl_3_): δ = 32.9 (s), 1.6 (s), −5.7 (s), −7.4 (s), −8.4 (s), −10.4 (br s) ppm.

## Results and Discussion

### Synthesis of HO–B^Me^oCb_2_ (**2**) and Generation of Me_3_Si–O–B^Me^oCb_2_ (**3**)

The reaction of BrB^Me^*o*Cb_2_ with Me_3_Si–OH forms HO–B^Me^oCb_2_ (**2**) in 85% yield, as shown in eq 2. The Me_3_Si–Br byproduct formed was detected in the ^1^H NMR spectra of reaction mixtures in CDCl_3_ (δ = 0.59 ppm). The ^1^H NMR spectrum of analytically pure **2** in CDCl_3_ solution contains signals at 2.04 ppm for the −CH_3_ on the carborane, and the ^11^B{^1^H} NMR spectrum contains a characteristic signal at 37.9 ppm for the central boron in **2**, consistent with formation of a tricoordinate boron in **2**.^34^ The solid-state ^11^B{^1^H} MAS NMR spectrum of **2** also contains a broad signal at 35 ppm for the tricoordinate boron.
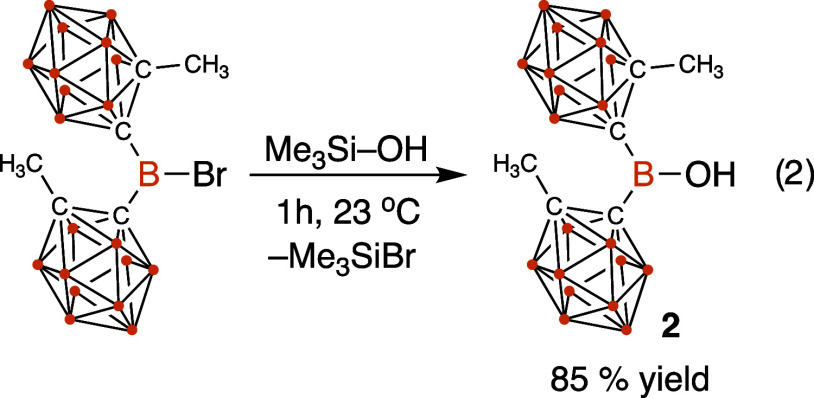
1a

**Figure 3:**
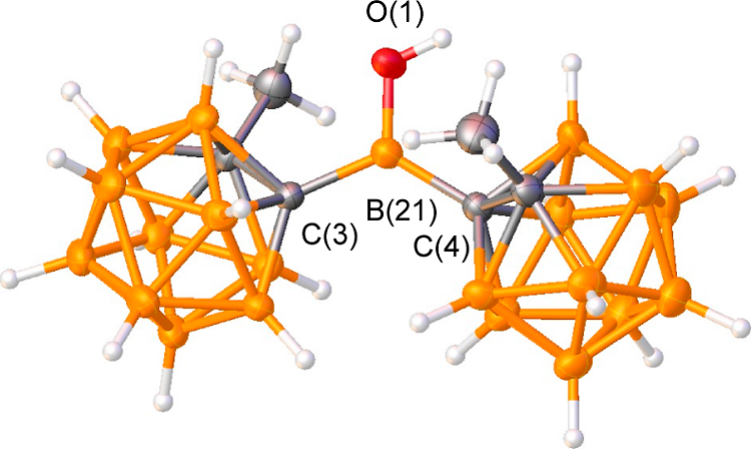
Solid-state structure of **2**. Thermal ellipsoids are drawn at the 50% probability level. Selected bond lengths (Å) and angles (deg): B(21)–C(3) 1.594(2), B(21)–C(4) 1.601(2), B(21)–O(1) 1.328(1), C(3)–B(21)–C(4) 126.1(1), C(3)–B(21)–O(1) 114.0(1), and C(4)–B(21)–O(1) 119.9(1).

The solid-state structure of **2** obtained from single-crystal X-ray diffraction data is shown in Figure 3. The sum of the bond angles around the central boron is 360°, indicating that **3** has a planar structure, which is consistent with the solution ^11^B{^1^H} NMR chemical shift mentioned above.

The reaction in eq 1 suggests that Me_3_Si–O–B^Me^oCb_2_ (**3**), which is a likely an intermediate in this reaction, is unstable in the presence of HBr. Preliminary results suggest that **3** is also rather unstable under standard silylation conditions. For example, **2** reacts with KH followed by Me_3_Si–OTf to form **3** in low yield in 95% purity as a viscous oil, eq 2. Attempts to purify **3** further were unsuccessful and yield **2** as the major product. **3** is sufficiently stable in CDCl_3_ solution to record NMR spectra. The ^11^B{^1^H} NMR spectrum of **3** contains a signal at 32.9 ppm that is consistent with a planar three-coordinate Lewis acidic boron in **3**.
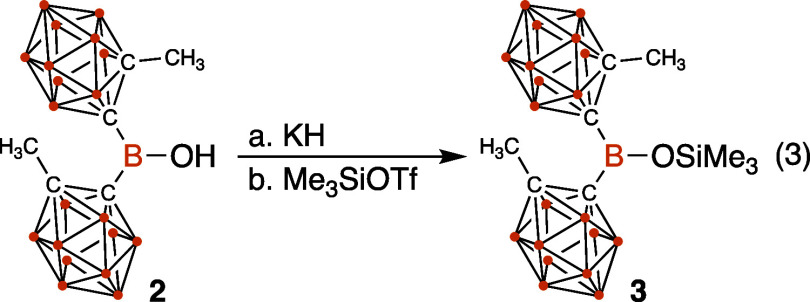
2

### Reaction of HB^Me^oCb_2_ with SiO_2-700_

**Figure 4:**
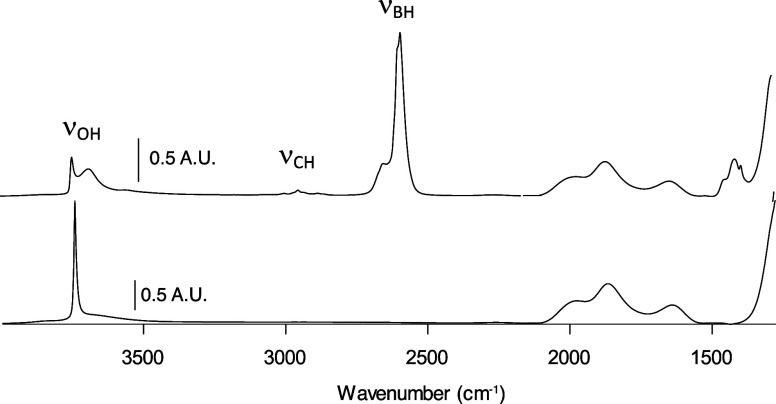
FTIR spectra of SiO_2-700_ (bottom) and **1** (top).

In benzene solution, HB^Me^*o*Cb_2_ reacts with SiO_2-700_ to form ^Me^*o*Cb_2_B(OSi≡) (**1**) and H_2_ (0.23 mmol g^–1^). ICP–OES of digested **1** gives 4.77 mmol_B_ g^–1^ (20.7 B/H_2_), which is close to the expected 21:1 ratio from the amount of H_2_ evolved in this reaction. Figure 4 shows the FTIR spectra of native SiO_2-700_ and **1**. The ν_OH_ values for isolated silanols in SiO_2-700_ decrease significantly in **1**, consistent with the reaction in Figure 2c, but unreacted silanols are present. Longer reaction times or an increase in the amount of HB^Me^*o*Cb_2_ do not significantly decrease the ν_OH_ band or increase boron loading from ICP–OES measurements. The FTIR spectrum of **1** contains broad ν_OH_ at 3686 cm^–1^, suggesting some type of hydrogen bonding interaction with residual silanols and the carborane groups. The spectrum for **1** also contains ν_CH_ at 2948 and 2878 cm^–1^ as well as ν_BH_ at 2649 and 2589 cm^–1^.

**Figure 5:**
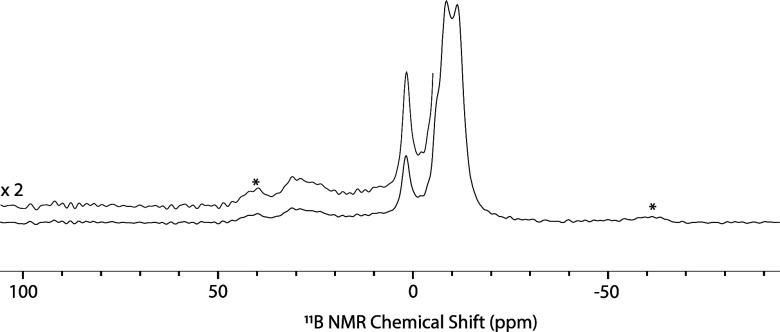
^11^B{^1^H} MAS NMR spectra of **1**. ν_rot_ = 10 kHz; * = spinning sideband.

The ^11^B{^1^H} MAS NMR spectrum of **1** is shown in Figure 5. A broad signal at 33 ppm is assigned to the central tricoordinate boron. Formation of tricoordinate boron sites is relatively common in boron-oxide-type materials,^15^ and these signals are typically broad due to the larger quadrupolar coupling (C_Q_) for tricoordinate boron compared that of to four-coordinate boron.^34^ Available ^11^B{^1^H} MAS NMR data suggests that supported B(C_6_F_5_)_3_ also adopts a tricoordinate structure.^23,35^ This is in contrast to the well-defined aluminum Lewis acid supported on silica mentioned above that forms a distorted tetrahedral aluminum site.^13^ The ^11^B{^1^H} MAS NMR spectrum also contains signals at 2, −6, −8, and −11 ppm for the borons that are part of the carborane dodecahedron. The ^13^C{^1^H} cross-polarization MAS (CPMAS) NMR spectrum of **1** contains the expected three signals at 25 (−CH_3_), 71, and 78 ppm (see the Supporting Information, Figure S13).

### Lewis Acidity of **1**

**Figure 6:**
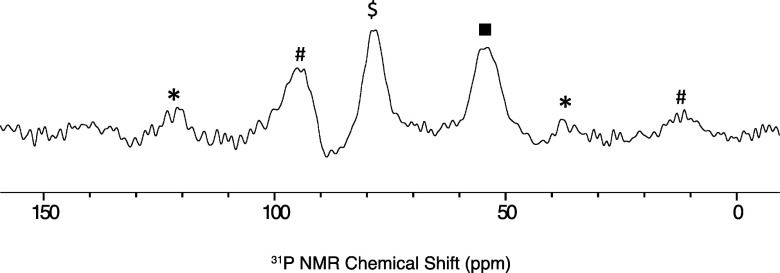
^31^P{^1^H} MAS NMR spectra of 1*TEPO. ν_rot_ = 10 kHz; $ = 1*TEPO; * = spinning sideband for 1*TEPO; black square = physisorbed TEPO; # = spinning side bands for physisorbed TEPO.

The change in ^31^P{^1^H} NMR chemical shift of TEPO is used as a diagnostic probe to determine Lewis acidity in solution^36^ or on solids containing Lewis acid sites.^37^ Contacting **1** with TEPO (1 equiv/B in **1**) results in the formation of the phosphine oxide adduct 1*TEPO, eq 3. The ^11^B{^1^H} MAS NMR spectrum of 1*TEPO contains a new signal at −1.5 ppm assigned to the tetrahedral central boron. Resonances for the boron atoms of the carborane groups appear at positions identical with those in **1**. However, this spectrum also contains minor residual signal intensity for the broad tricoordinate boron signal from **1** at 33 ppm, indicating that some Lewis acidic borons in **1** do not coordinate TEPO. The ^31^P{^1^H} MAS NMR data shown in Figure 6 is consistent with this observation, which contains a signal at 78 (Δδ = 28 ppm) and 54 ppm (Δδ = 4 ppm), assigned to the adduct and physisorbed TEPO, respectively. Attempts to generate TEPO adducts of **2** or **3** resulted in several ^31^P{^1^H} NMR signals in solution (Figures S9 and S10).
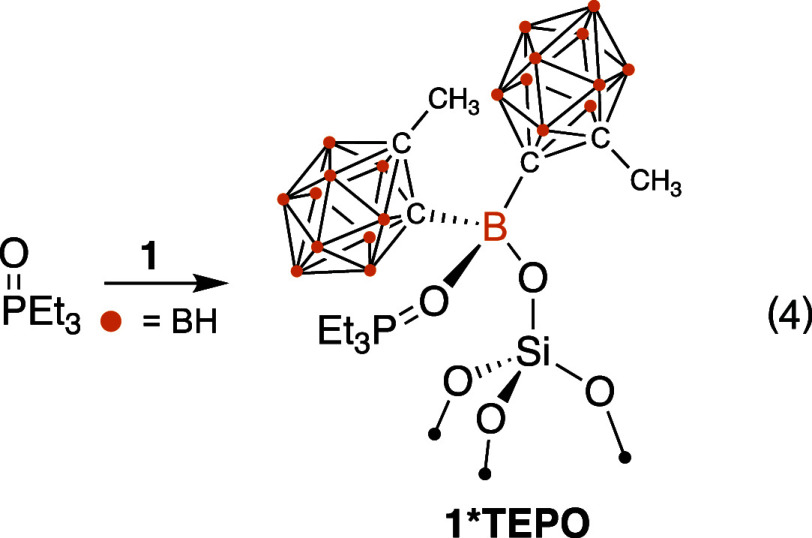
3

Selected ^31^P{^1^H} NMR data for the TEPO adducts are given in Table 1. The Δδ values obtained for 1*TEPO are similar to those obtained in solution for TEPO adducts of HB^Me^oCb_2_ and B(C_6_F_5_)_3_, indicating that the accessible boron in **1** is quite Lewis acidic. These values are identical with those obtained from the TEPO adduct of [≡SiOAl(OR^F^)_2_(O(Si≡)_2_)] but lower than the Δδ^31^P for the [Et_3_Si][**SZO**]. The latter surface species is a silylium-like ion, which are very strong Lewis acids that generally have large Δδ^31^P values.^38^ In solution, the Gutmann–Beckett method is unreliable for carboranyl boranes, exemplified with B*o*Cb_3_ being measured as less Lewis acidic than HB^Me^*o*Cb_2_ with ion affinities and other metrics indicating the opposite.^32^ This is due to the steric effects of the carborane groups.

**Table 1:** Selected Δδ ^31^P{^1^H} NMR Data, FIA, and HIA Data for Lewis Acids in Solution or Supported on Oxides

compound	Δδ	FIA kJ mol^–1^	HIA kJ mol^–1^	ref
**1**	28	493^a^	480^a^	this work
HB^Me^*o*Cb_2_	35.8 (C_6_D_6_) 30.0 (CDCl_3_)	527	540	(30)
B(C_6_F_5_)_3_	26.6 (CD_2_Cl_2_)	452	484	(39)
[≡SiOAl(OR^F^)_2_]	28	528	n.d.	(13)
[Et_3_Si][**SZO**]^b^	43	n.d.	n.d.	(40)

a Calculated for **4**, the DFT model of **1**.

b **SZO** = sulfated zirconium oxide; n.d. = not determined.

**Figure 7:**
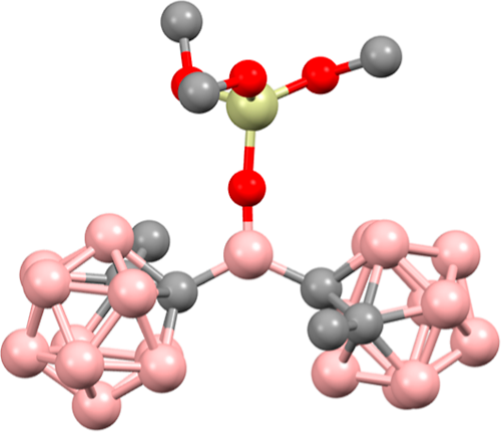
DFT structure of **4**. Selected bond lengths (Å) and angles (deg): B–C 1.629, B–O 1.337, Si–O 1.679, C–B–C 127.5, C–B–O 116.4, 116.1, C–Si–C 117.4, 107.5, 107.6, B–O–Si 174.5.

Two other common scales to assess Lewis acidity involve comparisons of density functional theory (DFT)-calculated fluoride ion affinity (FIA) or hydride ion affinity (HIA) values.^29,31^ Calculating the FIA or HIA of **1** is complicated by the complex amorphous silica surface but can be approximated by using a DFT-generated structure of (MeO)_3_Si–O–B^Me^*o*Cb_2_ (**4**), which contains a B–O–Si linkage that is similar to **1**. The structure of **4** optimized at the BP86/SVP level of theory is shown in Figure 7. **4** contains a planar central boron, and the bond lengths and angles about the central boron are close to those obtained experimentally from the XRD structure of **2**.

Values for the calculated FIA and HIA are also given in Table 1. **1** has an FIA of 493 kJ mol^–1^ and HIA of 480 kJ/mol^–1^. Compared to HB^Me^*o*Cb_2_ (FIA = 527 kJ mol^–1^; HIA of 540 kJ/mol^–1^), **4** is a weaker Lewis acid. By analogy, **1** is also predicted to be a weaker Lewis acid than HB^Me^*o*Cb_2_, which is reflected in the smaller Δδ value obtained using the Guttman–Beckett method. However, **4** is a stronger Lewis acid than B(C_6_F_5_)_3_ in terms of the FIA.

### Organometallic Reactivity of **1**

**Figure 8:**
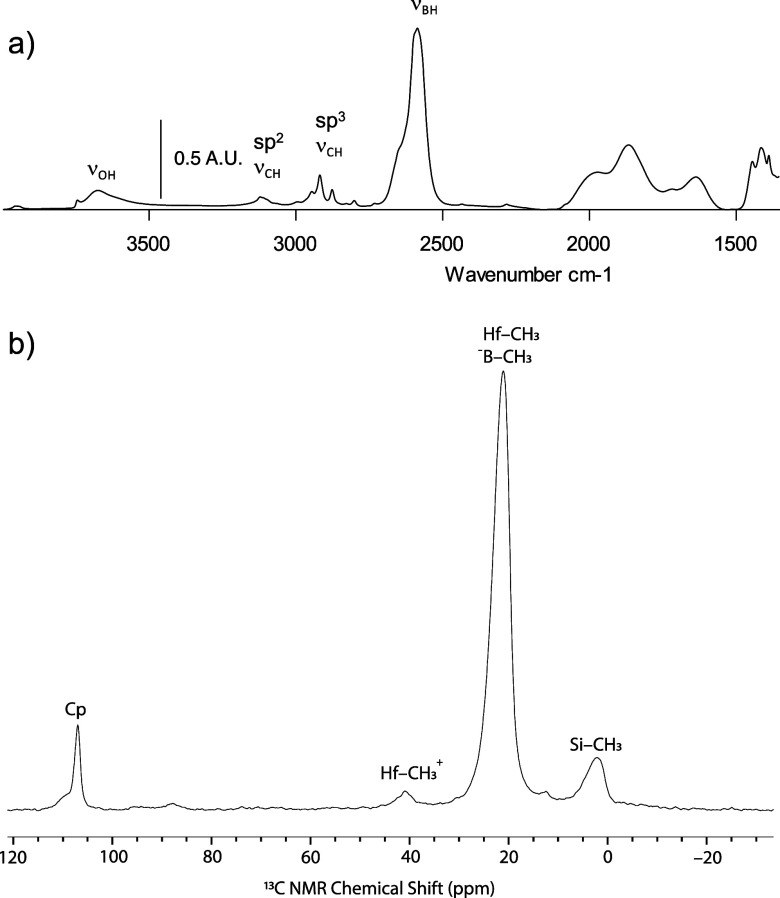
FTIR of Cp_2_Hf(^13^CH_3_)_2_/**1** (a). ^13^C{^1^H} CPMAS NMR spectrum of Cp_2_Hf(^13^CH_3_)_2_/**1**. ν_rot_ = 10 kHz (b).

Methide or hydride abstraction from an organometallic is a quintessential reaction of strong Lewis acid sites on oxides (Figure 1). To test if **1** is capable of any degree of methide abstraction, we treated the material with Cp_2_Hf(^13^CH_3_)_2_ (Cp = cyclopentadienyl), an organometallic known to react with well-defined aluminum Lewis acid, as shown in Figure 1b.^14^ This reaction results in the formation of CH_4_ (0.07 mmol g^–1^), indicating that Cp_2_Hf(^13^CH_3_)_2_ reacts with the residual silanols present on **1**. Indeed, FTIR data of **1** contacted with Cp_2_Hf(^13^CH_3_)_2_ shown in Figure 8a contains a reduced ν_OH_ band for silanols, consistent with their consumption. Also consistent with a surface reaction are the new sp^3^ C–H bands in this spectrum. The ν_BH_ band in this material is unperturbed with respect to that of **1**. ICP–OES analysis of the digested material gives 0.076 mmol_Hf_ g^–1^. This loading is surprisingly close to the amount of CH_4_ formed in this reaction, indicating that the major reaction pathway between **1** and Cp_2_Hf(CH_3_)_2_ involves silanols that do not react with HB^Me^*o*Cb_2_ on SiO_2-700_, resulting in the common protonolysis reaction shown in eq 1.

However, the ^13^C{^1^H} CPMAS NMR spectrum of **1** contacted with Cp_2_Hf(^13^CH_3_)_2_ shown in Figure 8b lends some tentative support for ionization by Lewis sites. Unsurprisingly, this spectrum contains an intense signal at 21 ppm for neutral Hf–^13^CH_3_ and a major Cp signal at 110 ppm. These results are consistent with the formation of Cp_2_Hf(^13^CH_3_)(OSi≡). This spectrum also contains a minor Cp signal at 113 ppm as well as a signal at 41 ppm for Hf–^13^CH_3_^+^, consistent with the formation of small amounts of [Cp_2_Hf–^13^CH_3_][H_3_^13^C–B(^Me^*o*Cb_2_)OSi≡]. A signal for the [H_3_^13^C–B(^Me^*o*Cb_2_)OSi≡] was not observed, which could be due to overlap with the Hf–CH_3_ signal in Cp_2_Hf(^13^CH_3_)(OSi≡).^41^ The signal at 2 ppm is from ≡Si–^13^CH_3_ sites. The ^11^B{^1^H} MAS NMR spectrum of **1** contacted with Cp_2_Hf(^13^CH_3_)_2_ contains minor additional signal intensity at −1 ppm (Figure S17). This is the expected region for a tetracoordinate boron, which was clearly observed in 1*TEPO.

**Scheme 1:**
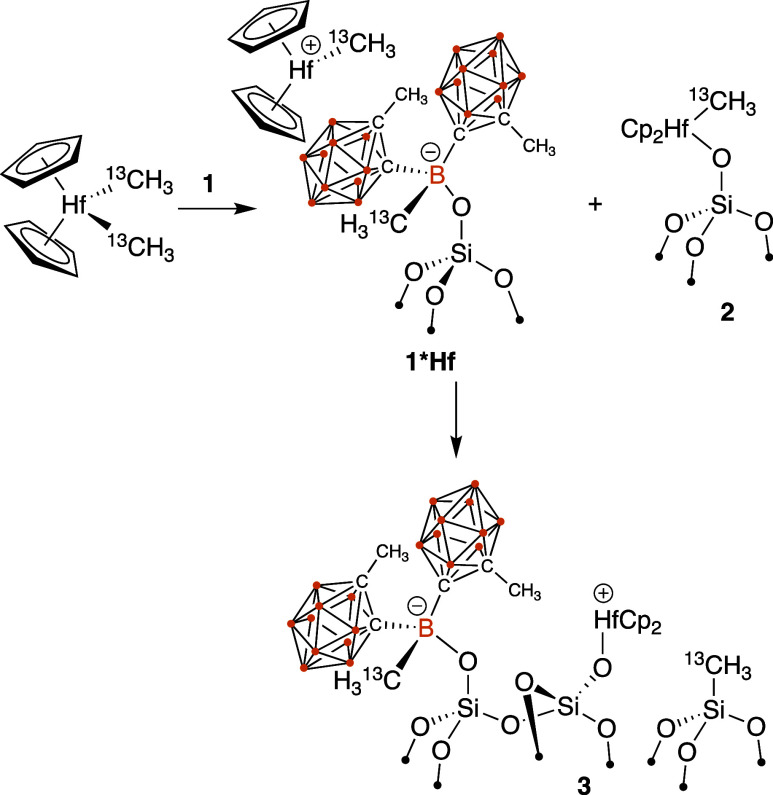
Reaction **1** with Cp_2_Hf(^13^CH_3_)_2_

The spectral data are consistent with those of the reactions in Scheme 1. Cp_2_Hf(^13^CH_3_)_2_ preferentially reacts with residual silanols present in **1** to form Cp_2_Hf(^13^CH_3_)(OSi≡) and methane. Cp_2_Hf(^13^CH_3_)_2_ also reacts with the boron Lewis sites in **1** to form [Cp_2_Hf–^13^CH_3_][H_3_^13^C–B(^Me^*o*Cb_2_)OSi≡ ]. Reactive d^0^ organometallic cations are known to react with silica surfaces by opening of Si–O–Si bridges by transferring alkyl groups and forming ≡Si–^13^CH_3_,^42^ which is consistent with the formation of **3**. However, the NMR signal intensities in both the ^13^C{^1^H} CPMAS NMR spectrum and the minimal signal intensity in the ^11^B{^1^H} MAS NMR spectrum for the tetrahedral [H_3_^13^C–B(^Me^*o*Cb_2_)OSi≡] anion indicate that the methide abstraction reaction in Scheme 1 is clearly a minor reaction pathway.

In a qualitative sense, the reactivity in Scheme 1 is remarkably similar to that obtained previously between Al(OC(CF_3_)_3_)(PhF)/silica and either Cp_2_Zr(CH_3_)_2_^13^ or Cp_2_Hf(CH_3_)_2_.^14^ However, **1** clearly forms less Hf–CH_3_^+^ than Al(OC(CF_3_)_3_)(PhF)/silica. Attempts to quantify the amount of [Cp_2_Hf–^13^CH_3_][H_3_C^13^–B(^Me^*o*Cb_2_)OSi ≡ ] formed on **1** using vinyl chloride as an active site probe by quantification of evolved propylene^43^ were consistent with very low surface coverage of the ion-pair (∼0.002 mmol g^–1^, see the Supporting Information for details). This result suggests that the sterically bulky carborane groups may restrict access to the central boron site in **1**, which results in low Hf–CH_3_^+^ surface coverage.

## Conclusions

There are limited examples showing that reactions of boranes and silica (or other oxides) form well-defined products and even fewer examples that form strong Lewis sites. Monomeric HB^Me^*o*Cb_2_ is a rare example, where a well-defined three-coordinate boron site forms when contacted with silica and the moderately strong Lewis acidity is preserved. This promising result suggests that other bulky secondary boranes may also react with oxides to form well-defined Lewis acid sites on the oxides. Tuning the steric environment in related boranes should result in a more efficient methide abstraction chemistry. However, this comes with the caveat that Lewis acidic boranes that would produce a more sterically open boron site often engage in monomer–dimer equilibria,^44^ which may affect the products obtained during surface functionalization chemistry.

## Supporting Information Available

Coordinates of 4 used for DFT calcuations (XYZ)
Experimental methods, solid-state NMR spectra, and gas chromatograph traces (PDF)

## Supplementary Material

ic3c04121_si_001.xyz
