# Supplementary material for: Formation of Strong Boron Lewis Acid Sites on Silica
Source: Inorg Chem. 2024 Mar 7;63(11):4939–46. doi: 10.1021/acs.inorgchem.3c04121 (PMC10951953; doi:10.1021/acs.inorgchem.3c04121)
Supplement: Supplementary file 2 — ic3c04121_si_002.pdf [file ic3c04121_si_002.pdf]

Supplementary Materials for:

Formation of Strong Boron Lewis Acid Sites on Silica

Kavyasripriya K. Samudrala,[1] Manjur O. Akram,[2] Jason L. Dutton,[3] Caleb D. Martin,[2]* and Matthew P. Conley[1]*

[1]Department of Chemistry, University of California, Riverside, California 92521, United States

[2]Department of Chemistry and Biochemistry, Baylor University, Waco, Texas 76798, United States

[3]Department of Biochemistry and Chemistry, La Trobe Institute for Molecular Science, La Trobe University, Melbourne, Victoria 3086, Australia

Email: matthew.conley@ucr.edu

Email: caleb_d_martin@baylor.edu

Table S1. X-ray crystallographic details for $HOB^{Me}oCb_2$.

	**$HOB^{Me}oCb_2$**
**CCDC**	2330796
**Empirical Formula**	$C_6H_{27}B_{21}O$
**FW (g/mol)**	342.28
**Crystal System**	orthorhombic
**Space Group**	*P bca*
**a (Å)**	15.1757(6)
**b (Å)**	13.3018(4)
**c (Å)**	20.3436(8)
**α (deg)**	90
**β (deg)**	90
**γ (deg)**	90
**V (Å^3)**	4106.6(3)
**Z**	8
**D_c (g cm^{-3})**	1.107
**Radiation λ (Å)**	0.71073
**Temp (K)**	150
**R1 [I>2(σ)I]^a**	0.0552
**wR2 (F^2)^a**	0.1704
**GOF (S)^a**	1.072

^{a} $R1(F[I > 2(I)]) = \sum||F_o| - |F_c||/\sum|F_o|$; $wR2(F^2\,[\text{all data}]) = \{[w(F_o^2 - F_c^2)2]/[w(F_o^2)^2]\}^{1/2}$; $S(\text{all data}) = [w(F_o^2 - F_c^2)^2/(n - p)]^{1/2}$ (n = no. of data; p = no. of parameters varied; $w = 1/\sigma^2(F_o^2) + (aP)^2 + bP]$ where $P = (F_o^2 + 2F_c^2)/3$ and a and b are constants suggested by the refinement program.

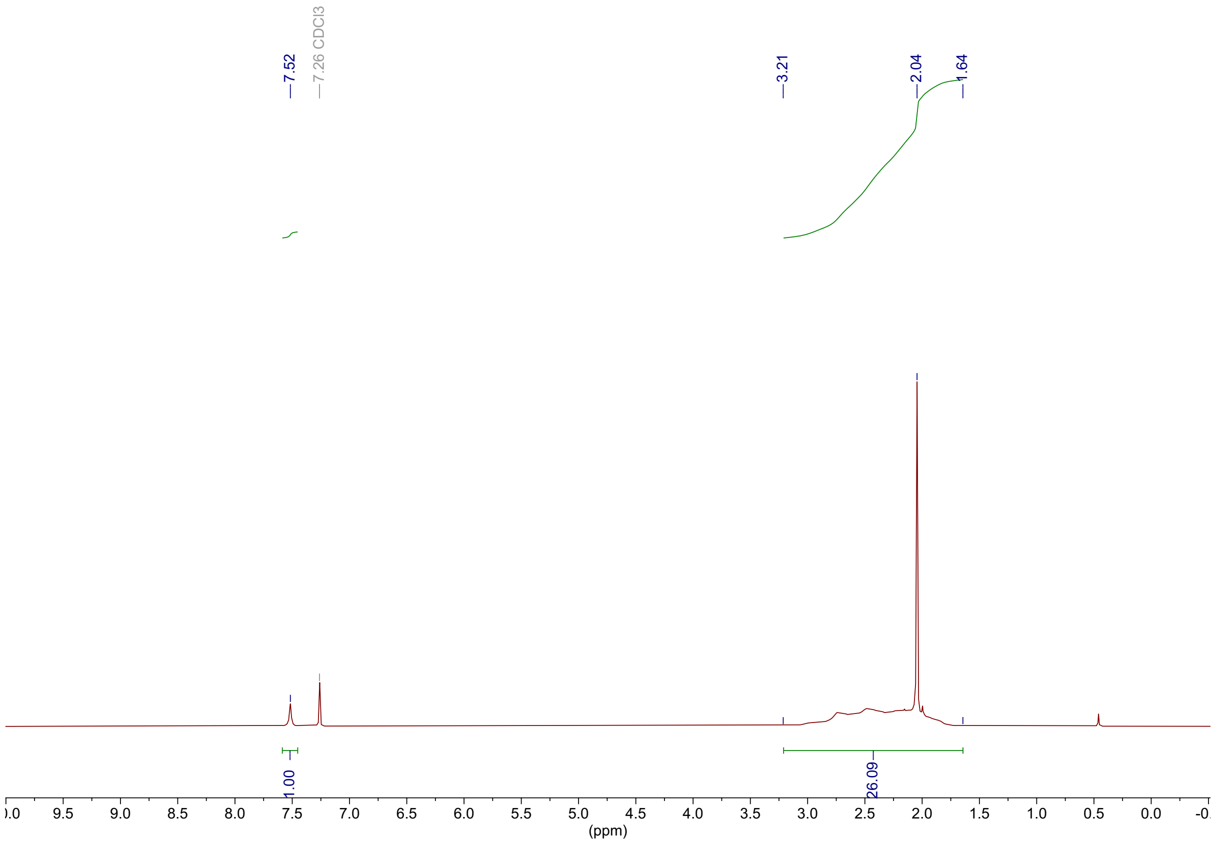

Figure S1. ^{1}H NMR spectrum of $HOB^{Me}oCb_2$ in $CDCl_3$ (600 MHz)

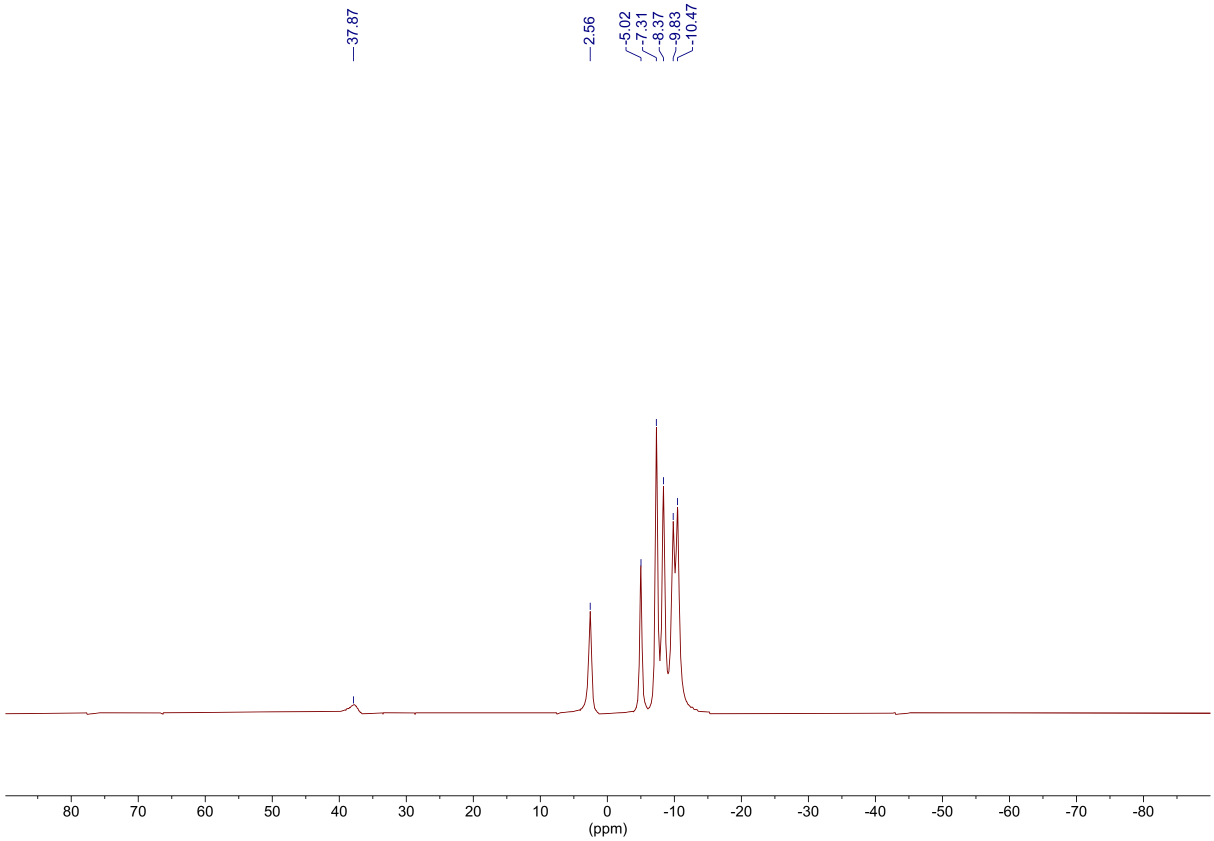

Figure S2. $^{11}B\{^{1}H\}$ NMR spectrum of $HOB^{Me}o Cb_2$ in $CDCl_3$ (193 MHz)

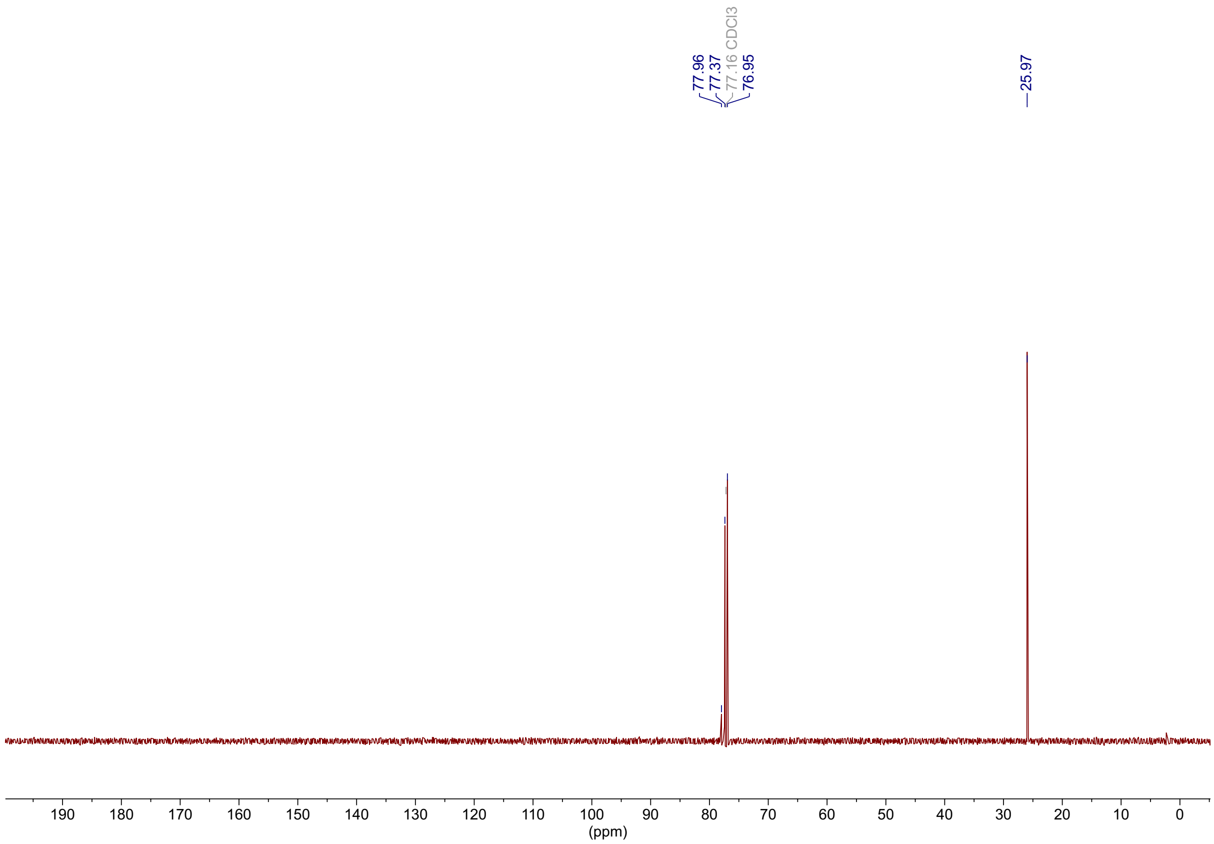

Figure S3. $^{13}C\{^{1}H\}$ NMR spectrum of $HOB^{Me}oCb_2$ in $CDCl_3$ (151 MHz)

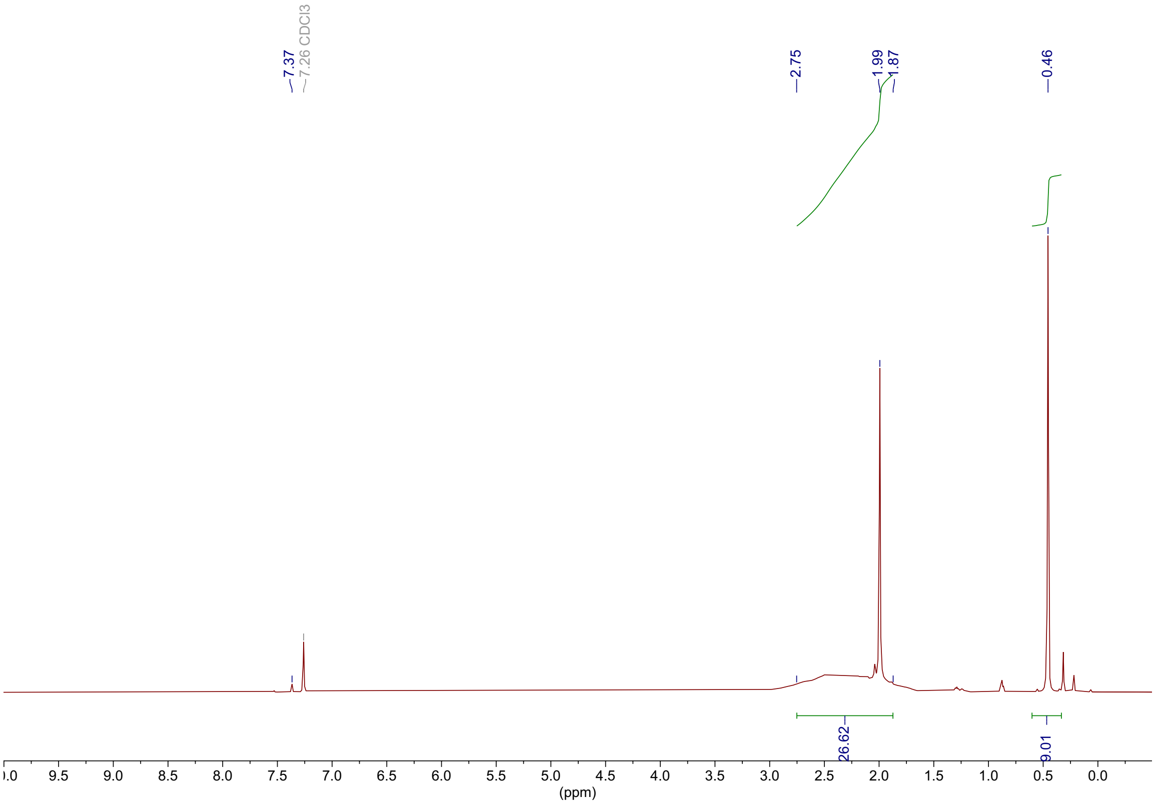

Figure S4. ^{1}H NMR spectrum of Me_3SiOB^{Me}*o*Cb_2 in $CDCl_3$ (600 MHz)

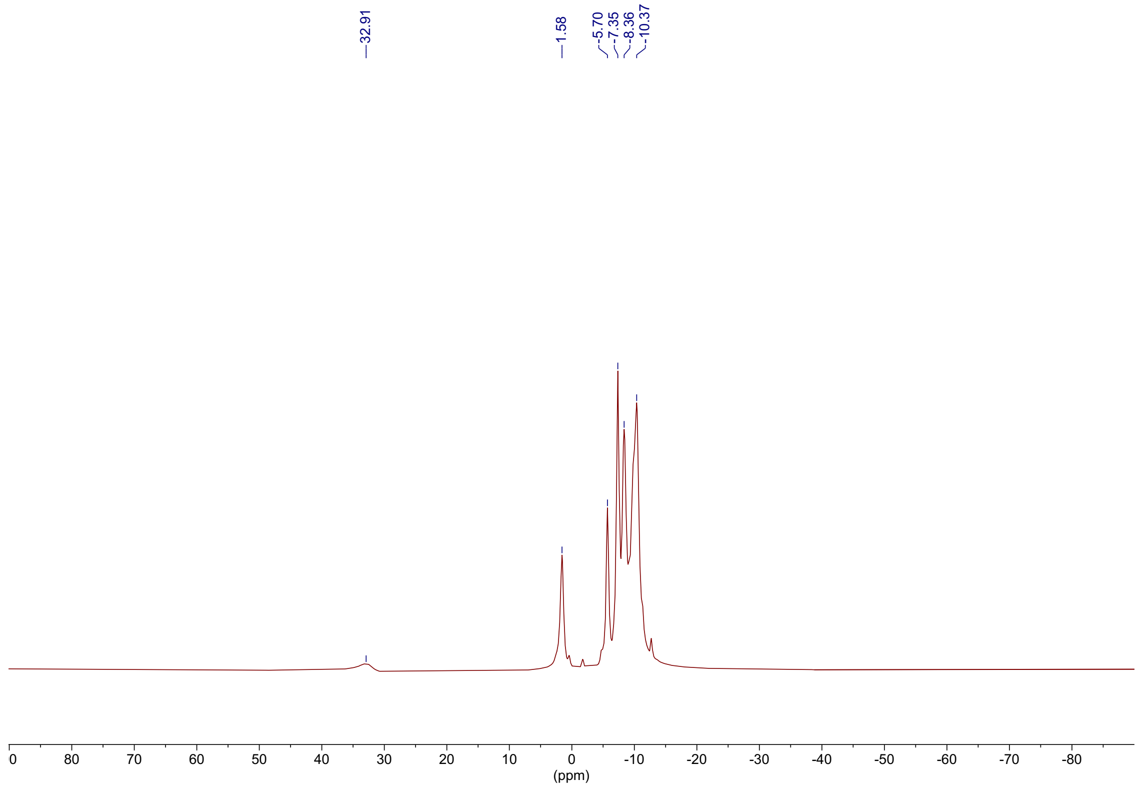

Figure S5. $^{11}B\{^{1}H\}$ NMR spectrum of $Me_3SiOB^{Me}\textit{o}Cb_2$ in $CDCl_3$ (193 MHz)

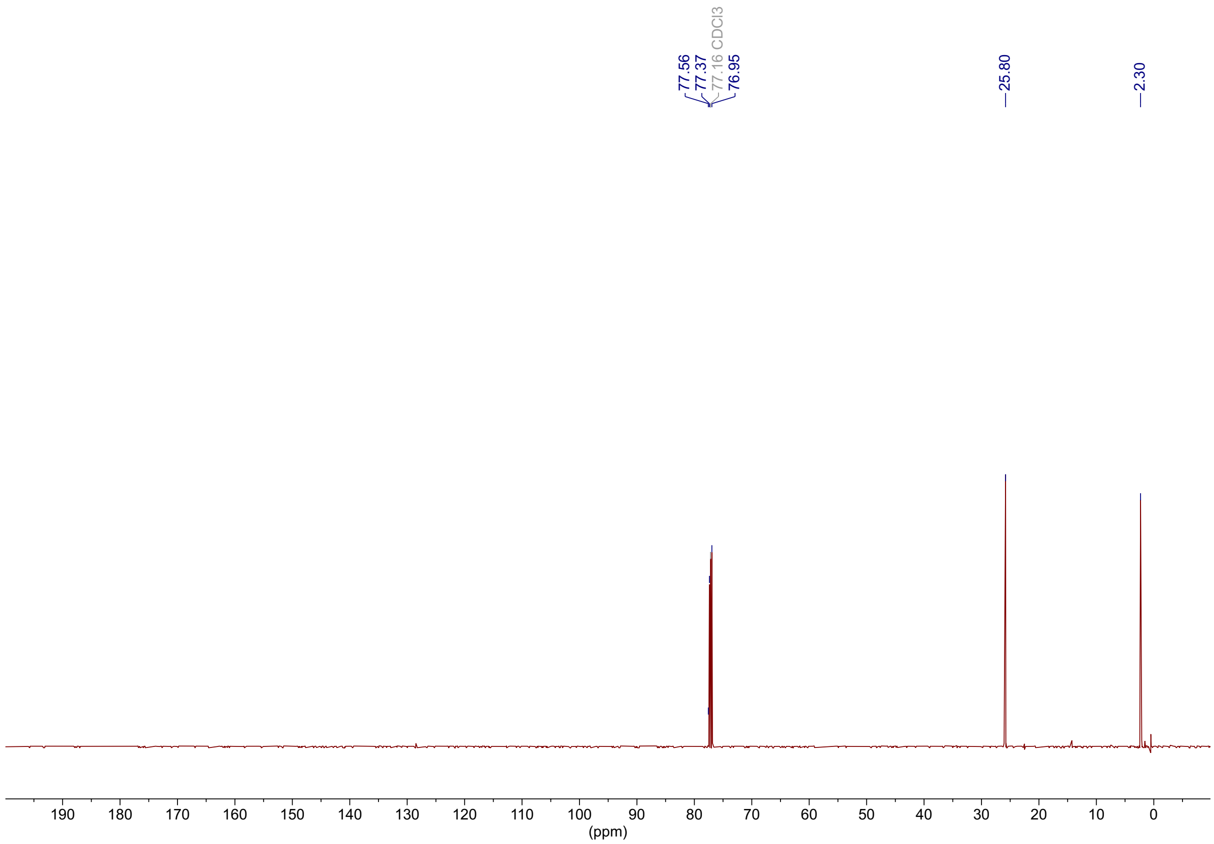

Figure S6. $^{13}C\{^1H\}$ NMR spectrum of $Me_3SiOB^{Me}o Cb_2$ in $CDCl_3$ (151 MHz)

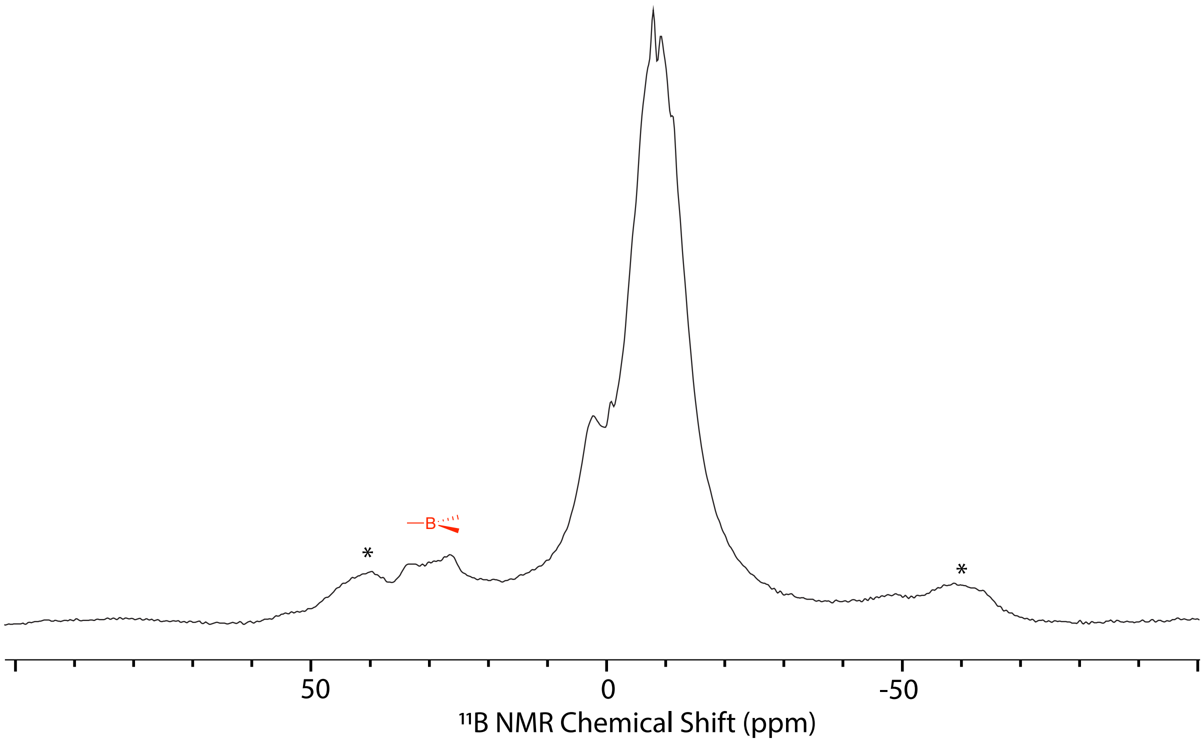

Figure S7. 10 kHz $^{11}B\{^{1}H\}$ MAS NMR of **2** acquired at room temperature and 10 kHz spinning speed. The signal for the tricoordinate boron at 35 ppm is labelled. Spinning sidebands are labelled with a "*," other signals in this spectrum are from the carborane.

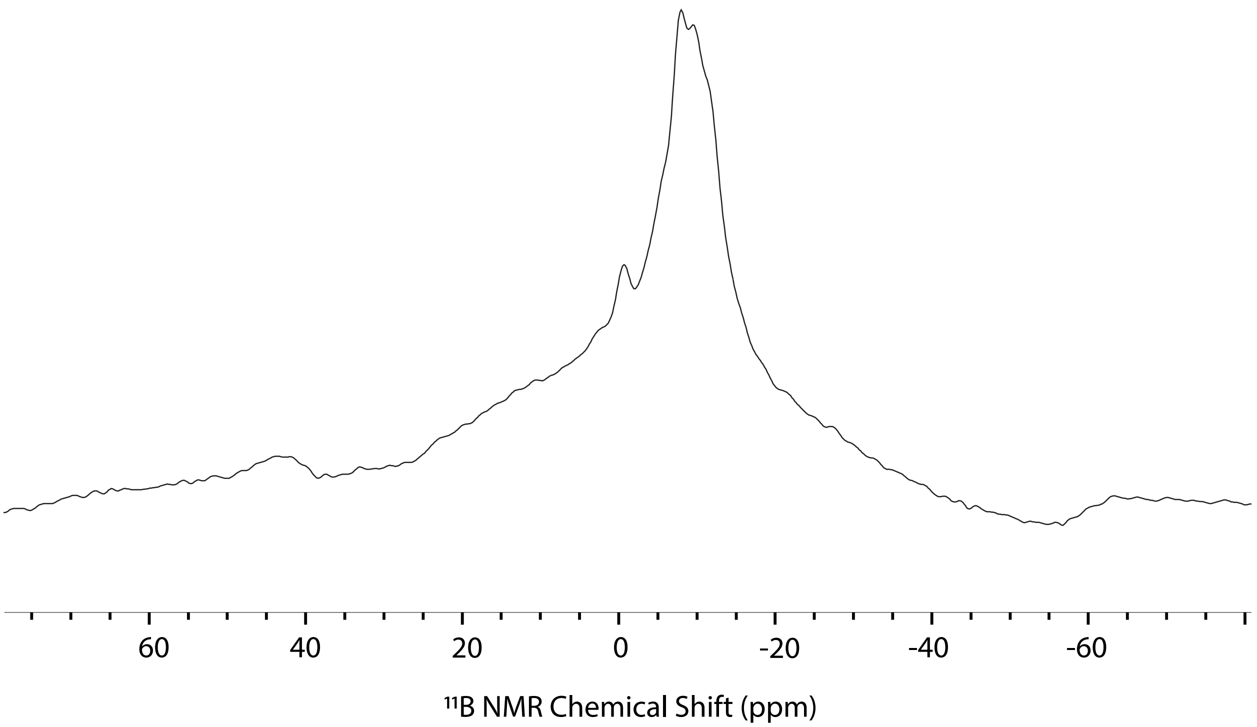

Figure S8. 10 kHz $^{11}B\{^{1}H\}$ MAS NMR of $HB^{Me}oCb_2$ acquired at room temperature and 10 kHz spinning speed.

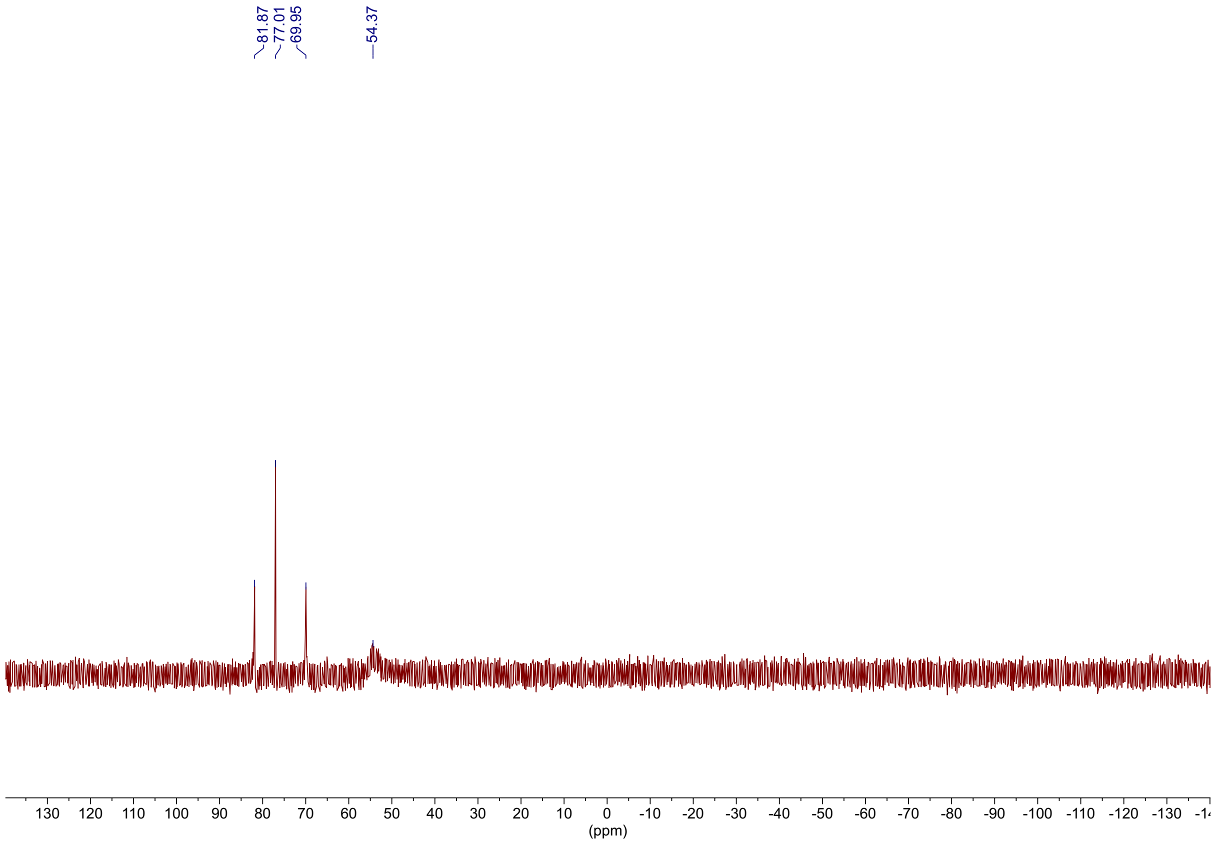

Figure S9. $^{31}P\{^{1}H\}$ NMR spectrum of the equimolar reaction of HOB^{Me}*o*Cb_2 with $OPEt_3$ in $CDCl_3$ (243 MHz)

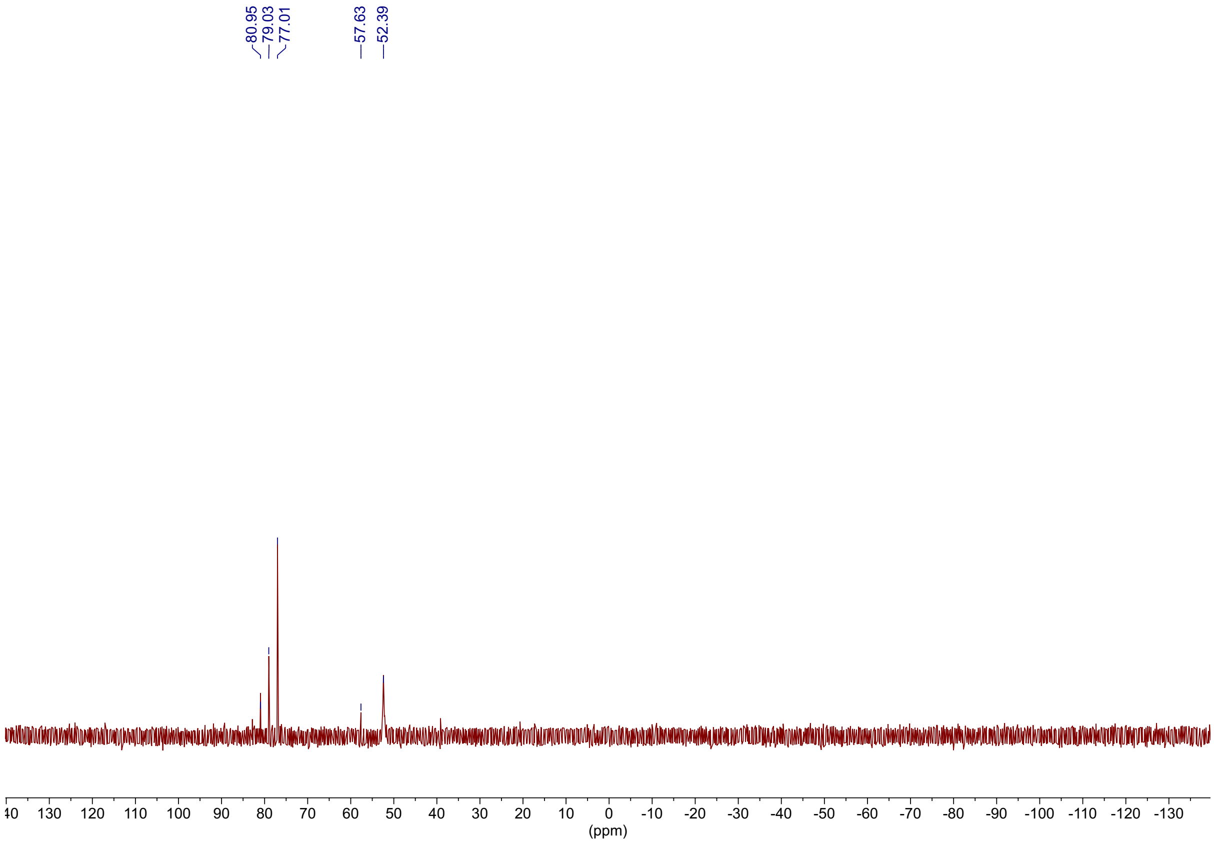

Figure S10. $^{31}P\{^1H\}$ NMR spectrum of the equimolar reaction of Me_3SiOB^{Me}*o*Cb_2 with $OPEt_3$ in $CDCl_3$ (243 MHz)

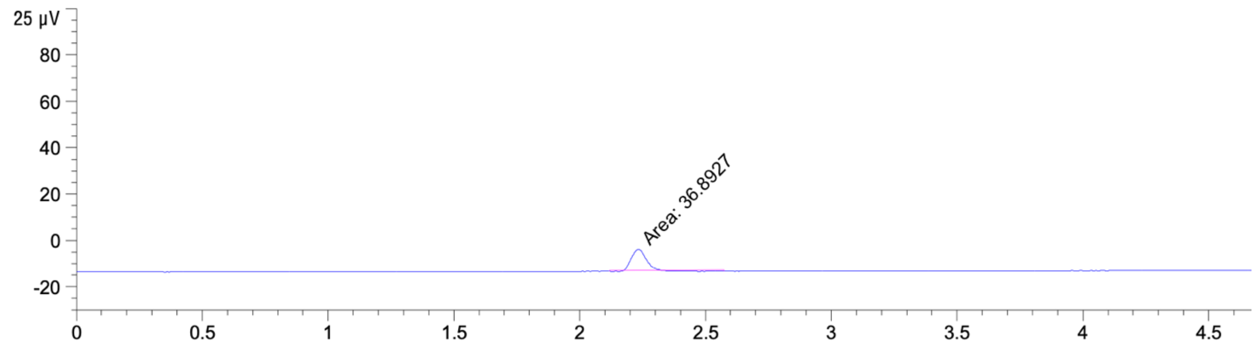

Figure S11. GC-TCD of grafting volatiles from **H-BSO**, 0.23 mmol H_2/g SiO_2.

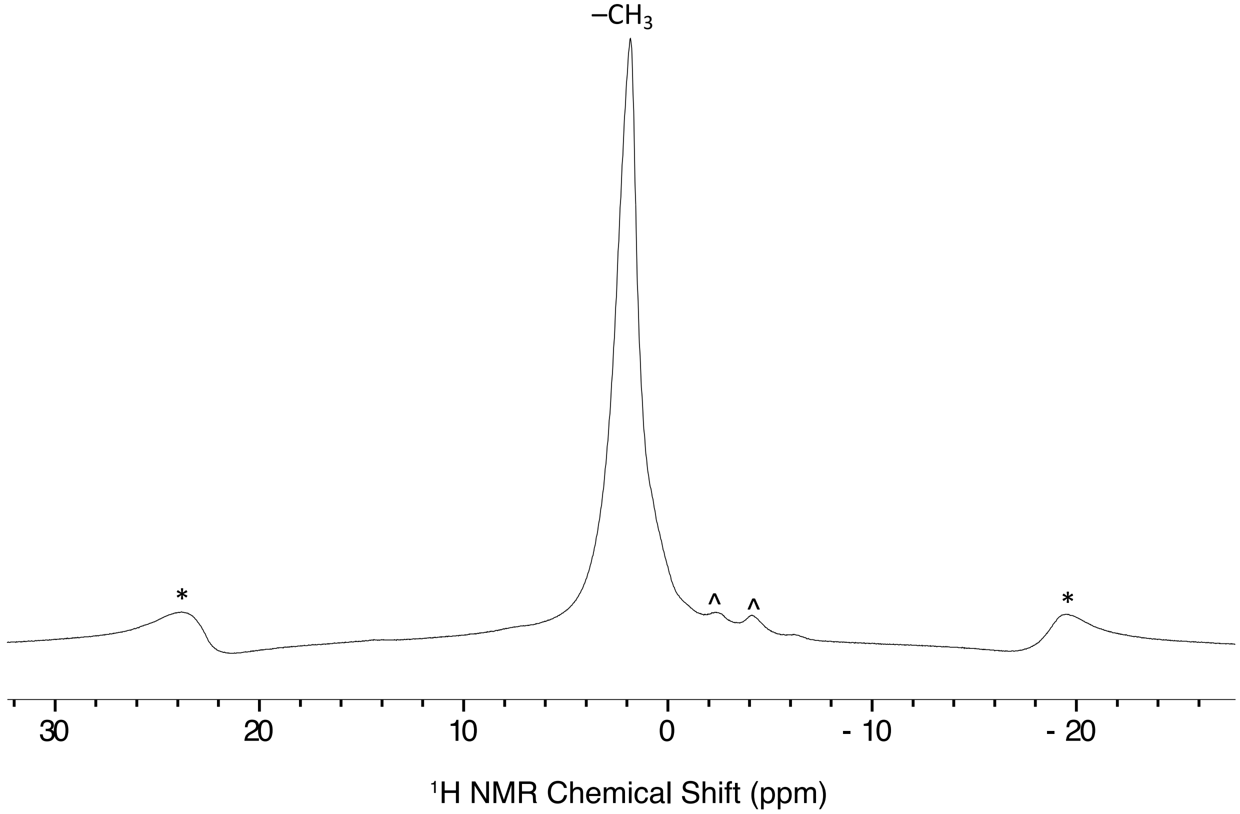

Figure S12. 12.5 kHz ^{1}H MAS NMR of **1** acquired at room temperature, 1.86 ppm (≡Si-O–B(**Me***o*Cb)$_2$). Signals labeled with ^ at -2.4 ppm and -4.1 ppm are from probe background. * = spinning sideband

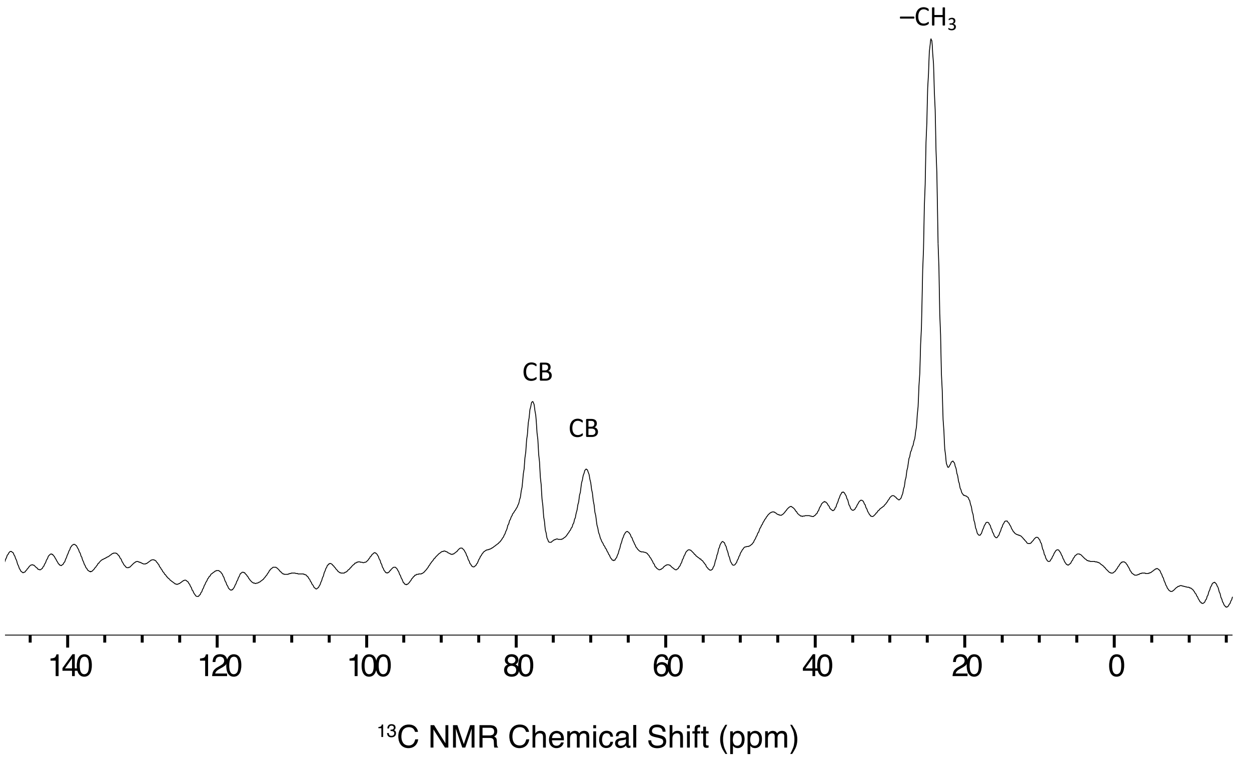

Figure S13. 10 kHz ^{13}C CPMAS NMR of **1** acquired at room temperature containing signals at 24.6 ppm (≡Si-O-B(**Me**_o_Cb)$_2$), 70.8 and 77.8 ppm (≡Si-O-B(Me_o_**Cb**)$_2$).

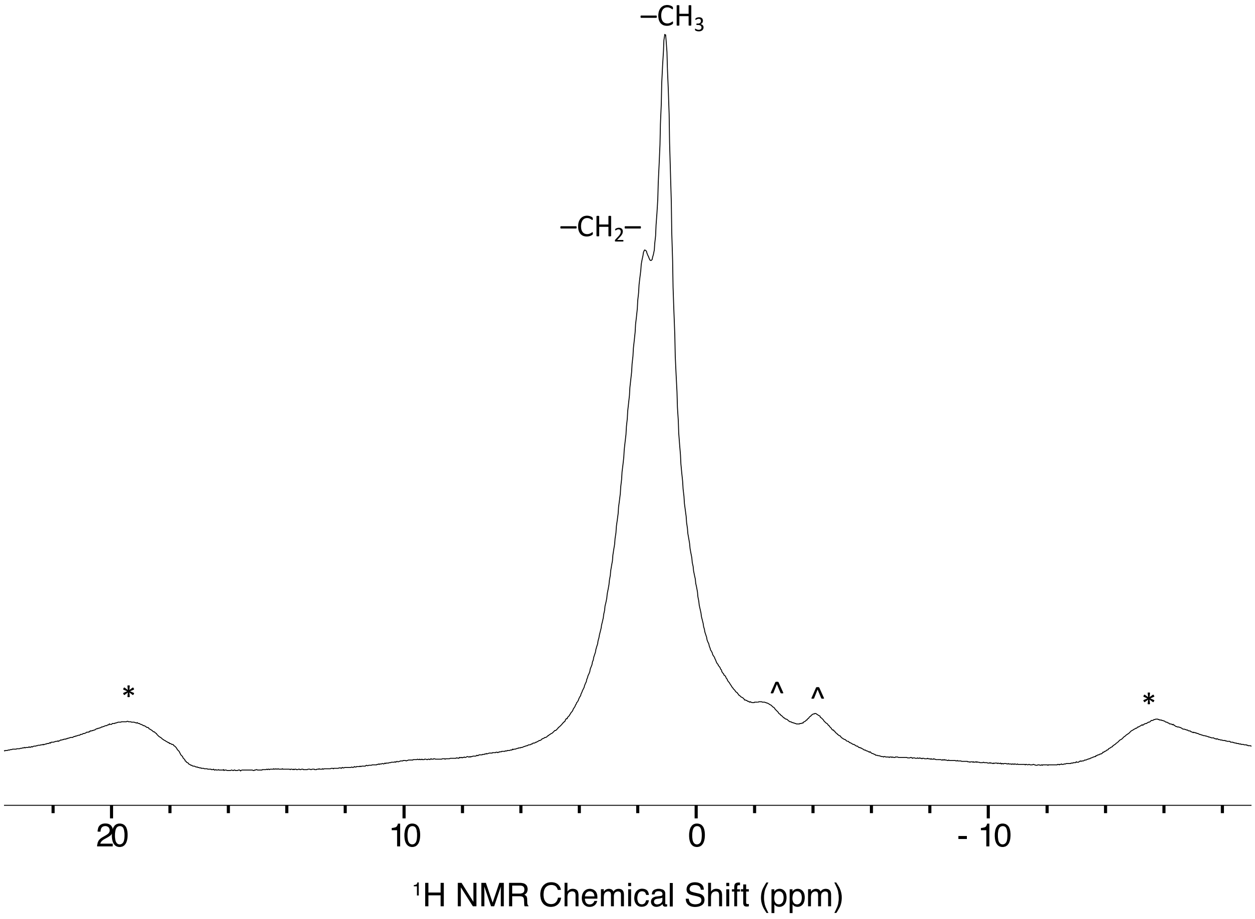

Figure S14. 10 kHz ^{1}H MAS NMR of **1*TEPO** acquired at room temperature containing signals at 1.1 ppm (C**H_3**CH_2)$_3$P=O) 1.7 ppm (CH_3C**H_2**)$_3$P=O). Signals labeled with ^ at -2.4 ppm and -4.1 ppm are from probe background. * = spinning sideband

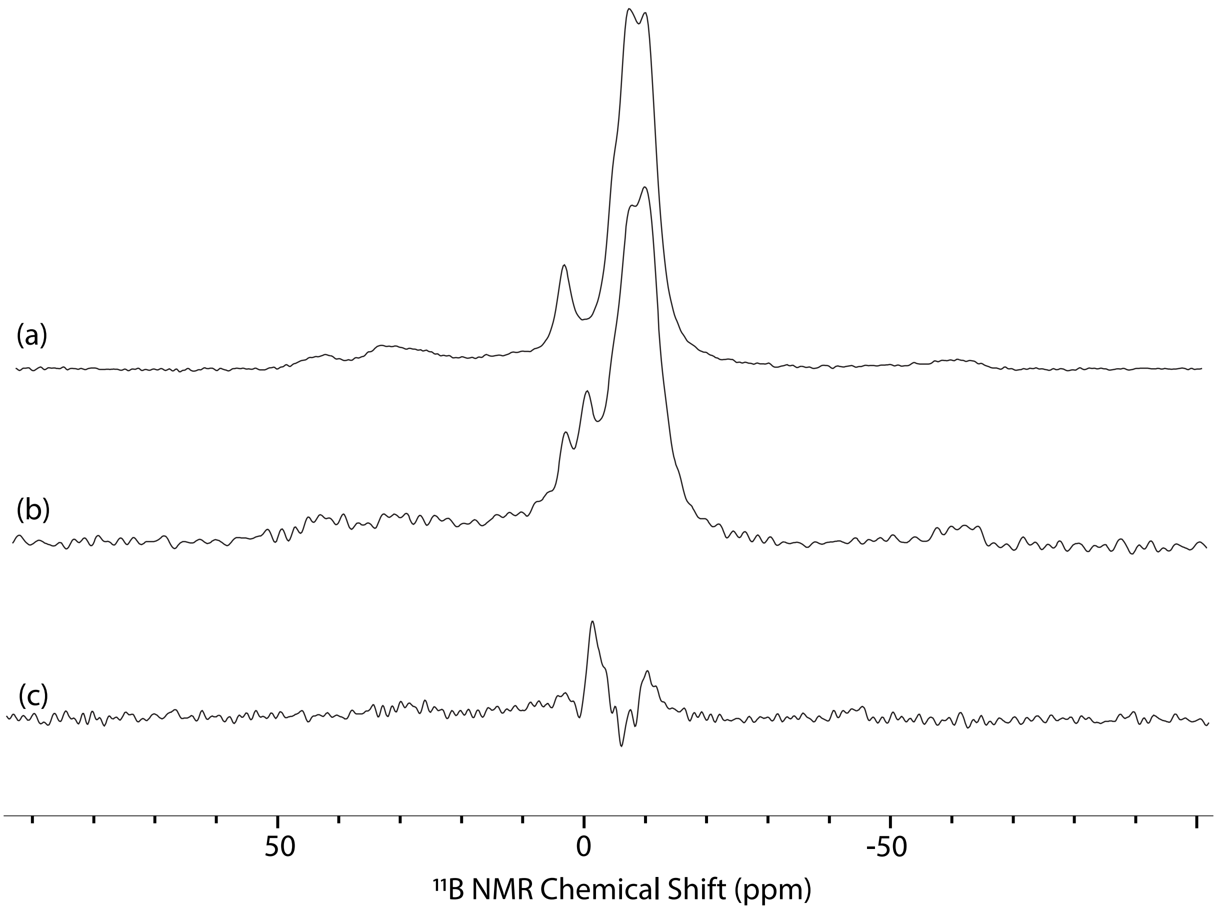

Figure S15. 10 kHz MAS Hahn echo $^{11}B\{^1H\}$ MAS NMR of **1** (a) and **1*TEPO** (b) acquired at room temperature. The difference spectrum is shown in (c), and contains a signal at -1.5 ppm assigned to the tetrahedral boron in **1*TEPO**. The spectrum in (b) contains a small amount of singal intensity at 33 ppm (≡Si-O-**B**(Me*o*Cb)$_2$), indicating that not all Lewis acidic boron reacts with TEPO.

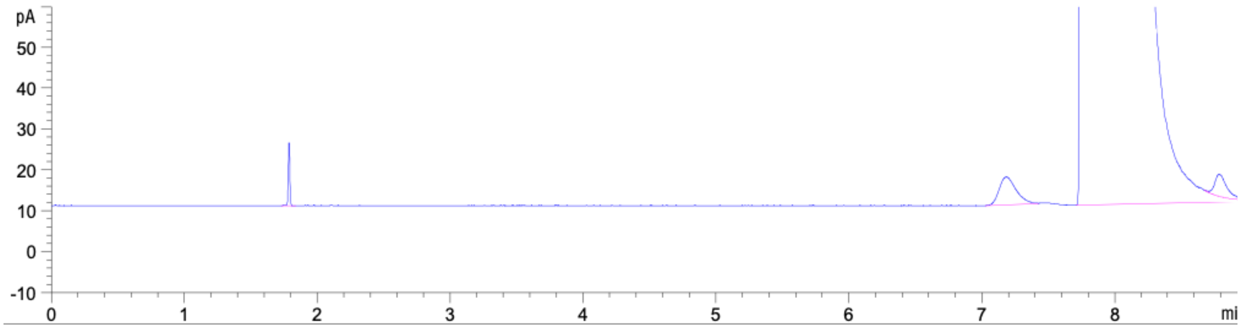

Figure S16. GC-FID of volatiles released during grafting of Cp_2HfMe_2 on **1**, 0.07 mmol CH4/g SiO2

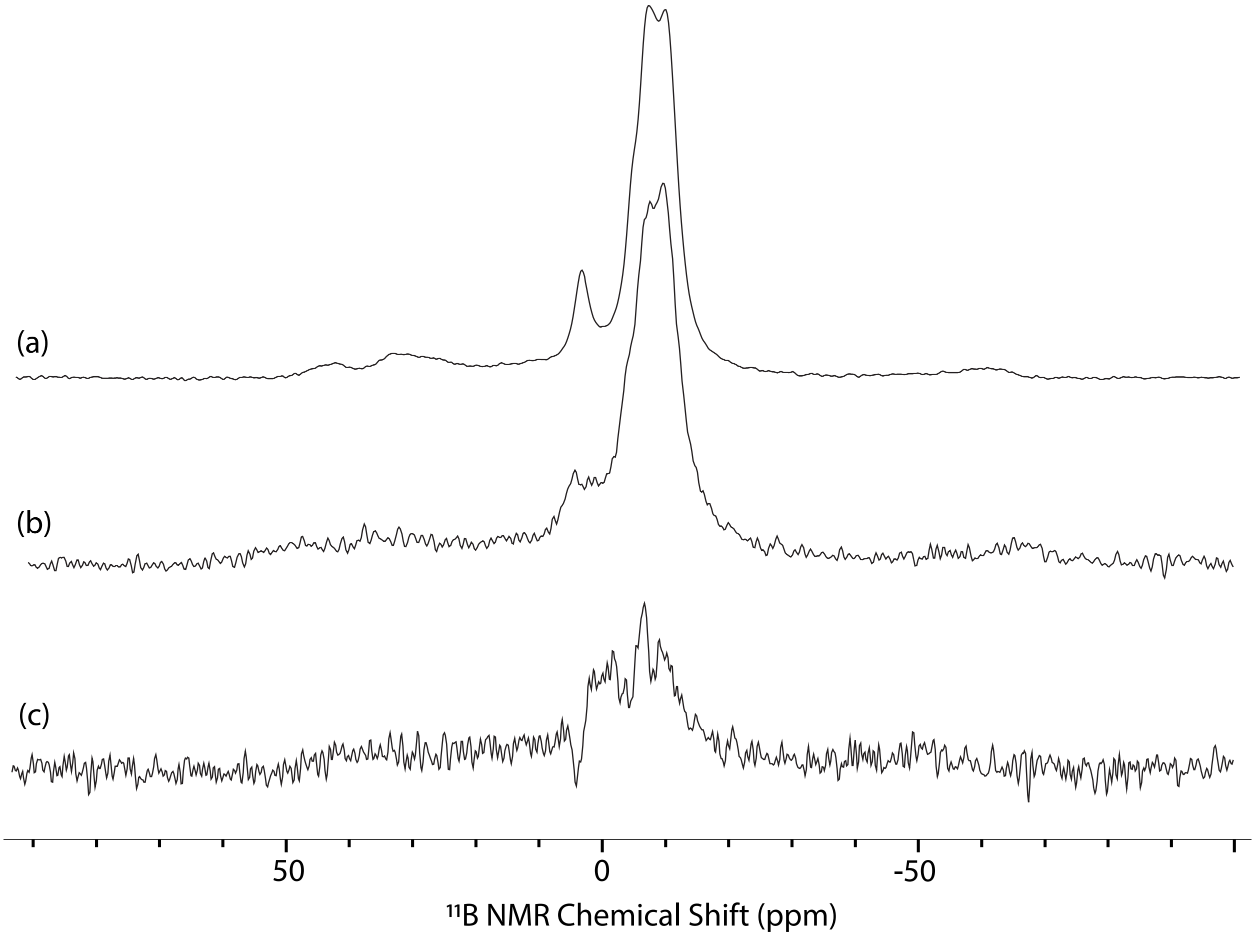

Figure S17. 10 kHz MAS Hahn echo ^{11}B NMR of **1** and **1*Hf** (b). The difference spectrum is shown in (c) and contains minor, but detectable, signal intensity at -1.5 ppm assigned to the tetrahedral boron in **1*Hf**.

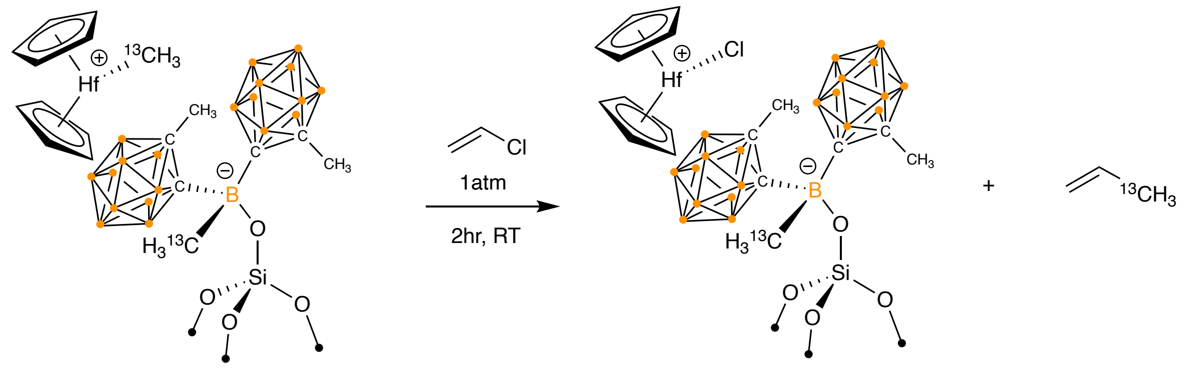

Reaction of Vinyl Chloride with 1*Hf: 0.100 g **1*Hf** was transferred into a 100 mL round bottom flask fitted with a Teflon tap fitted high vacuum adapter an argon-filled glovebox. The flask was removed from the glovebox, connected to a high vacuum line, and evacuated for 5 min. 1 atm of vinyl chloride, dried rigorously over molecular sieves and freshly generated BASF copper catalysts to remove water and oxygen, was added to the flask. The flask was left at room temperature for 2 hours. The volatiles were quantified by GC-FID, showing that 0.002 mmol propylene/g SiO_2 are released during this reaction.

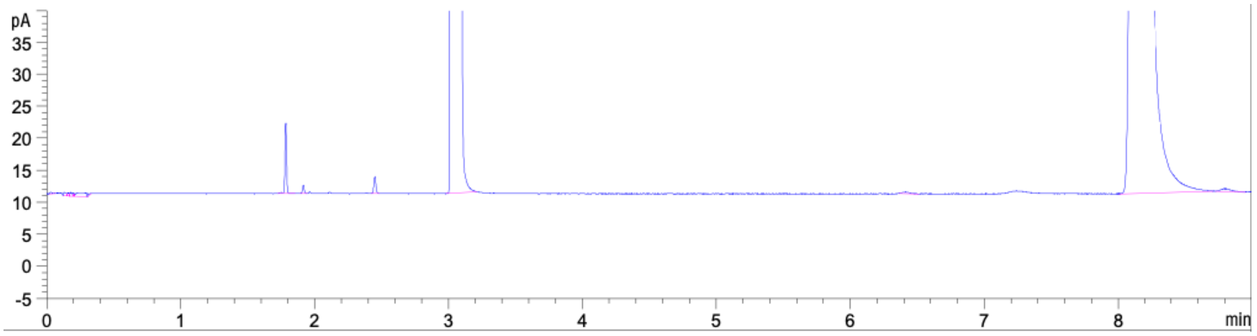

Figure S18. GC-FID of volatiles released after contacting vinyl chloride with Hf/H-BSO, 0.002 mmol propylene/g SiO_2

DFT Calculations were performed using Gaussian16.[1] Coordinates and enthalpies are given for the BP86/SV(P) geometry optimizations and single-point vibrational frequency calculations.[2] Fluoride and hydride affinities were calculated using Krossing's method, which uses an isodesmic comparison to the fluoride and hydride affinity of $[(H_3C)_3Si]+$.[3]

Model Borane

Enthalpy: -1475.244858 Hartree

C	0.00000000	0.00000000	0.00000000
C	-0.69486700	-0.09860800	1.35263700
H	-1.72311800	-0.49295300	1.25355000
H	-0.73116500	0.88593200	1.85613600
H	-0.12523000	-0.79344300	1.99808900
C	-0.78466100	0.77663000	-1.27013000
B	0.55207500	1.56506600	-0.49998300
H	0.31179600	2.48430300	0.23896600
B	1.69361000	0.27097400	-0.07516700
B	0.98043900	-1.28229600	-0.58656600
H	1.07880900	-2.25787000	0.11591900
B	2.23584600	-0.50544600	-1.59317900
B	1.41371500	0.32154700	-2.95023200
H	1.90294000	0.45550800	-4.04707900
B	0.79983100	-1.26002600	-2.36168600
B	-0.60362000	-0.94562100	-1.31956600
H	-1.56100400	-1.63721500	-1.13026200
B	-0.34618800	0.05068200	-2.77238200
B	0.37763400	1.59728100	-2.26595700
H	0.03817800	2.62637200	-2.79035600
B	1.96855600	1.26752100	-1.52935900
H	2.86005200	2.08261800	-1.57150000
H	-1.17486800	0.02112600	-3.64758500
H	0.84192600	-2.27853400	-3.00922400
H	3.34055000	-0.98767200	-1.67905800
H	2.27681900	0.35607800	0.97772000
B	-2.20685600	1.55312500	-1.10506100
C	-3.69567000	0.89512800	-1.05139100
C	-4.73318500	1.21174000	-2.34097500
C	-4.18991000	1.99316500	-3.53197500
H	-4.96489900	2.02474400	-4.32104000
H	-3.29423200	1.50190900	-3.95904000
H	-3.93477200	3.03116800	-3.24529900
B	-4.14448900	-0.40529600	-2.09499300
B	-5.46759000	-1.26572100	-1.27001200
B	-6.83545500	-0.12357100	-1.07904400

H -7.98766300 -0.48572200 -1.11891100
B -5.75009600 -0.44826000 0.30562000
B -4.09895600 -0.62148600 -0.33178300
H -3.22157000 -1.28494800 0.14918400
B -4.58997000 0.91152500 0.42690400
B -4.98207300 2.07018300 -0.86125600
H -4.70407000 3.23594500 -0.84501900
B -6.29421600 1.22753400 -0.02637700
H -7.02823800 1.85006500 0.70433300
B -6.34770900 1.43817500 -1.79795800
H -7.02262700 2.22846200 -2.41067200
B -5.83348600 -0.08849500 -2.56149900
H -6.15985100 -0.34301900 -3.69454200
H -4.02599800 1.31462300 1.41647100
H -6.08807500 -1.05563600 1.29433200
H -5.60318200 -2.45333600 -1.44227600
H -3.32722800 -0.82123600 -2.86974700
O -2.14395000 2.88717500 -1.04840000
Si -1.92599300 4.55188000 -1.05953900
O -3.15417700 5.05293200 -2.05269200
C -3.23461900 6.34865000 -2.65026000
H -4.00000800 6.30949300 -3.45106600
H -3.54397400 7.11608300 -1.90722900
H -2.26301000 6.65223000 -3.09537600
O -0.51253700 5.06695200 -1.71684700
C 0.73577600 5.34958900 -1.08540700
H 1.18057800 6.24005800 -1.57522400
H 0.60971900 5.56069200 -0.00251000
H 1.43157400 4.49386200 -1.21172400
O -1.96999700 5.06895000 0.50734100
C -2.97596500 4.84011600 1.48990100
H -2.67929500 5.37297000 2.41580700
H -3.96608000 5.22546900 1.16275400
H -3.08288200 3.75863000 1.72541200

Model Borane Fluoride

Enthalpy: -1575.176963 Hartree

C 0.00000000 0.00000000 0.00000000
C -0.51554300 -0.37571200 1.38562900
H -1.53563300 -0.79593400 1.33459600
H -0.54146100 0.50354100 2.05222000
H 0.16143000 -1.14013800 1.81351300
C -1.01261500 0.81285100 -1.11263800
B 0.30649800 1.65433100 -0.36035900

H	0.05994600	2.47432000	0.48506400
B	1.63666200	0.47855400	-0.17520400
B	1.06349900	-1.08470900	-0.80018400
H	1.33667700	-2.11044000	-0.22117000
B	2.12730400	-0.07061600	-1.80472500
B	1.09918600	0.78073700	-2.98965700
H	1.47718600	1.08149200	-4.10153900
B	0.71574900	-0.90811000	-2.53526900
B	-0.61702600	-0.85864900	-1.34847000
H	-1.44987000	-1.69741800	-1.15177600
B	-0.59855600	0.29203400	-2.70073300
B	-0.01427200	1.84591000	-2.09196900
H	-0.47702500	2.87896100	-2.49592000
B	1.66213700	1.63898900	-1.52071600
H	2.45166200	2.55845600	-1.54365700
H	-1.46753600	0.23954400	-3.53347000
H	0.81560300	-1.84709100	-3.29415500
H	3.27091100	-0.40652700	-2.02801700
H	2.30730700	0.52383500	0.83052800
B	-2.54058200	1.42454400	-0.59898100
C	-3.95401000	0.48129200	-0.86748200
C	-4.89987100	0.66407100	-2.26428600
C	-4.41321600	1.55775900	-3.39994400
H	-4.99158500	1.31085100	-4.31127900
H	-3.34190700	1.38561300	-3.60978500
H	-4.54883600	2.62670700	-3.15417900
B	-4.08583900	-0.82328700	-1.99771000
B	-5.31689500	-1.94087000	-1.35348400
B	-6.87722700	-1.05605600	-1.29425900
H	-7.94332200	-1.60327900	-1.48113500
B	-5.91006800	-1.26195300	0.19381200
B	-4.18373000	-1.11138300	-0.24908900
H	-3.26214300	-1.64697700	0.31331500
B	-5.02354700	0.25969300	0.49026500
B	-5.43098600	1.39854100	-0.80082800
H	-5.38129200	2.59529500	-0.71768800
B	-6.68688000	0.31407800	-0.15260900
H	-7.59802200	0.77053300	0.50373500
B	-6.57857600	0.59717100	-1.90505200
H	-7.31281000	1.29023800	-2.57049600
B	-5.74379700	-0.78826700	-2.64156100
H	-5.90227500	-1.04331000	-3.81281200
H	-4.68795100	0.68709400	1.56418900
H	-6.25457400	-1.96704400	1.11831100
H	-5.22890600	-3.12859000	-1.57599200
H	-3.15293100	-1.09022000	-2.70016500

O	-2.71716400	2.71183700	-1.22673700
Si	-2.86368200	4.32709700	-1.09197200
O	-4.16280400	4.72220700	-2.07870500
C	-4.50817900	6.05955000	-2.38535700
H	-5.36869700	6.05050700	-3.08857200
H	-4.81160100	6.63334000	-1.47801400
H	-3.66450800	6.60520200	-2.86676200
O	-1.59411100	5.22187600	-1.70052600
C	-0.41881200	5.58680000	-0.99795000
H	0.00859300	6.49863300	-1.47008400
H	-0.62797100	5.80940700	0.07241600
H	0.34853000	4.78227300	-1.04757200
O	-3.05733300	4.91243700	0.46026500
C	-3.86453800	4.37781600	1.49888100
H	-4.94912000	4.42615900	1.24783500
H	-3.60231700	3.32152700	1.71871000
H	-3.69872200	4.98573700	2.41497600
F	-2.45331100	1.54960900	0.82132800

Model Borane Hydride

Enthalpy -1475.936057 Hartree

C	0.00000000	0.00000000	0.00000000
C	-0.58100000	-0.23487700	1.38925400
H	-1.60675900	-0.64238200	1.33378500
H	-0.60997400	0.70322700	1.97298700
H	0.05867000	-0.96590500	1.92086500
C	-0.95028700	0.77670600	-1.18459100
B	0.39239100	1.60577800	-0.46213700
H	0.15533800	2.48873800	0.32297000
B	1.66232300	0.38864800	-0.16330000
B	1.02544900	-1.18599200	-0.69382900
H	1.23775800	-2.18118100	-0.04028300
B	2.15915800	-0.29254900	-1.73798500
B	1.19834600	0.52253600	-3.00443200
H	1.61735300	0.72880100	-4.12329000
B	0.72699100	-1.11210400	-2.44700600
B	-0.63003400	-0.92078900	-1.30300800
H	-1.50710200	-1.70516300	-1.07352400
B	-0.52552100	0.13091800	-2.72819200
B	0.11319100	1.69522200	-2.20832800
H	-0.29641300	2.72064100	-2.68497300
B	1.76853600	1.45340000	-1.58325300
H	2.60126900	2.33193800	-1.64803100
H	-1.37916500	0.06117700	-3.57591800

H	0.80146200	-2.10532500	-3.13674700
H	3.29003100	-0.69465000	-1.91154100
H	2.31140900	0.47160200	0.85431900
B	-2.41969000	1.48206800	-0.69653000
C	-3.87967200	0.64253400	-0.92776500
C	-4.82861100	0.84241600	-2.32356300
C	-4.28686500	1.67128800	-3.48365100
H	-4.89096000	1.45308000	-4.38573900
H	-3.23329300	1.41427000	-3.69656400
H	-4.33695400	2.75136200	-3.25593800
B	-4.11484900	-0.68561100	-2.01753800
B	-5.41118900	-1.69583600	-1.32577800
B	-6.90796900	-0.70503900	-1.28061600
H	-8.01095000	-1.18375300	-1.43983500
B	-5.94098300	-0.92965700	0.20526900
B	-4.21284100	-0.91065400	-0.25932700
H	-3.32603800	-1.49284800	0.31300000
B	-4.94789500	0.53583200	0.44059700
B	-5.29024900	1.65827800	-0.87913200
H	-5.16162500	2.85124000	-0.83690800
B	-6.61203500	0.68310600	-0.18455200
H	-7.48277200	1.22258000	0.46388500
B	-6.50243000	0.90372200	-1.94723400
H	-7.19626100	1.62489400	-2.62620600
B	-5.77467600	-0.55925300	-2.64709300
H	-5.96612300	-0.84011100	-3.80762500
H	-4.55666900	0.97712500	1.49414900
H	-6.32101900	-1.58119400	1.15503100
H	-5.40686600	-2.89376100	-1.50783100
H	-3.20582400	-1.03955800	-2.71445300
O	-2.51513700	2.78983600	-1.34976700
Si	-2.50525400	4.39731000	-1.13628200
O	-3.82571100	4.95893700	-2.00830400
C	-4.04387900	6.33088900	-2.27129300
H	-4.94325100	6.42974400	-2.91704900
H	-4.22686800	6.91532100	-1.33823200
H	-3.17874800	6.79570900	-2.79751800
O	-1.20470600	5.21951000	-1.78173200
C	0.03364800	5.47893900	-1.14584500
H	0.48268100	6.39035800	-1.59915800
H	-0.08808000	5.65458000	-0.05337000
H	0.74733700	4.63727100	-1.28954900
O	-2.54662300	4.90354800	0.46168500
C	-3.44838600	4.44261700	1.45449800
H	-3.19553700	4.93915000	2.41693700
H	-4.50595800	4.69301100	1.20721900

H -3.38368700 3.34216900 1.60107700
H -2.34110100 1.56848500 0.53369700

Fluoride enthalpy: -99.682216 Hartree

Hydride enthalpy: -0.486377 Hartree

References

(1) M. J. Frisch, G. W. T., H. B. Schlegel, G. E. Scuseria, M. A. Robb, J. R. Cheeseman, G. Scalmani, V. Barone, G. A. Petersson, H. Nakatsuji, X. Li, M. Caricato, A. Marenich, J. Bloino, B. G. Janesko, R. Gomperts, B. Mennucci, H. P. Hratchian, J. V. Ortiz, A. F. Izmaylov, J. L. Sonnenberg, D. Williams-Young, F. Ding, F. Lipparini, F. Egidi, J. Goings, B. Peng, A. Petrone, T. Henderson, D. Ranasinghe, V. G. Zakrzewski, J. Gao, N. Rega, G. Zheng, W. Liang, M. Hada, M. Ehara, K. Toyota, R. Fukuda, J. Hasegawa, M. Ishida, T. Nakajima, Y. Honda, O. Kitao, H. Nakai, T. Vreven, K. Throssell, J. A. Montgomery Jr., J. E. Peralta, F. Ogliaro, M. Bearpark, J. J. Heyd, E. Brothers, K. N. Kudin, V. N. Staroverov, T. Keith, R. Kobayashi, J. Normand, K. Raghavachari, A. Rendell, J. C. Burant, S. S. Iyengar, J. Tomasi, M. Cossi, J. M. Millam, M. Klene, C. Adamo, R. Cammi, J. W. Ochterski, R. L. Martin, K. Morokuma, O. Farkas, J. B. Foresman and D. J. Fox. Gaussian 16. **2016**.

(2) a) Becke, A. D. Density-functional exchange-energy approximation with correct asymptotic behavior. *Phys. Rev. A* **1988**, *38*, 3098-3100; b) Schäfer, A.; Horn, H.; Ahlrichs, R. Fully optimized contracted Gaussian basis sets for atoms Li to Kr. *J. Chem. Phys.* **1992**, *97*, 2571-2577.

(3) Bohrer, H.; Trapp, N.; Himmel, D.; Schleep, M.; Krossing, I. From unsuccessful H2-activation with FLPs containing B(Ohfip)3 to a systematic evaluation of the Lewis acidity of 33 Lewis acids based on fluoride, chloride, hydride and methyl ion affinities. *Dalton Trans.* **2015**, *44*, 7489-7499.
